# Operando Investigation of Mechanochemically Synthesized Ni‐Based Metal–Organic Frameworks for Electrocatalytic Alcohol Oxidation

**DOI:** 10.1002/cssc.202502581

**Published:** 2026-04-21

**Authors:** Arkendu Roy, Xavier Vaneberck, Celia Ganado Rodriguez, Semen Butyrin, Dominik Al‐Sabbagh, Chayanika Das, Klas Meyer, Ines Feldmann, Jörg Radnik, Ana Guilherme Buzanich, Franziska Emmerling, Biswajit Bhattacharya

**Affiliations:** ^1^ Federal Institute of Materials Research and Testing (BAM) Berlin Germany; ^2^ Humboldt University Berlin Germany

**Keywords:** alcohol oxidation reaction, electrocatalysis, mechanochemistry, metal–organic framework, operando investigation

## Abstract

A nickel‐based metal–organic framework (Ni‐MOF) with mixed N‐ and O‐donor linkers was synthesized via both mechanochemical and solvothermal routes and evaluated as an electrocatalyst for alcohol oxidation reactions (AORs). Despite having identical crystal and electronic structures, the mechanochemically synthesized Ni‐MOF (termed as MC‐MOF) exhibited markedly superior catalytic activity compared to its solvothermally synthesized analog (ST‐MOF). Structural characterization confirmed that enhanced performance arises from the distinct morphology and higher density of accessible Ni active sites in MC‐MOF. Specifically, the MC‐MOF exhibited a roughly 4.4‐fold higher electrochemically active surface area, enabling the highly stable, selective oxidation of various alcohols to valuable products over 24 h of continuous operation. Operando quick X‐ray absorption spectroscopy revealed that under alkaline conditions, MC‐MOF undergoes potential‐dependent structural evolution, displaying distinct catalytic pathways for oxygen evolution and AORs. While Ni centers oxidize to higher valence states during the oxygen evolution reaction, they remain largely in the Ni^2+^ state during AORs, indicating selective suppression of high‐valent Ni‐oxygenated species in the presence of alcohol molecules in the electrolyte medium. This study demonstrates that mechanochemical synthesis can effectively tailor the morphology and catalytic behavior of MOF‐based electrocatalysts, offering an environmentally benign and scalable route for developing advanced materials for sustainable energy conversion.

## 1 Introduction

Mechanochemical synthesis, a process involving chemical reactions induced by mechanical energy, is promising as an environmentally benign and economical method for catalytic materials synthesis [1, 2, 3, 4]. Conventional wet chemistry methods are often considered hazardous due to the large amount of solvent waste they produce, and the multistep synthesis process makes them difficult to scale up for a given formulation of catalytic material [5, 6]. In recent years, however, mechanochemical synthesis has shown great potential for synthesizing various materials, such as nanoparticles, oxides, MOFs, COFs, and high‐entropy materials, on a large scale [7, 8, 9]. Mechanochemically synthesized catalysts differ fundamentally from those synthesized using conventional solvent‐based methods. The underlying reasons for differences in catalytic activity, whether structural, morphological, or due to defect sites, are not yet fully understood. An important area of active research remains identifying key factors that specifically enhance or alter catalytic performance in mechanochemically synthesized materials, such as particle size distribution, surface chemistry, crystallinity, and the presence of unique defect structures [2, 10].

Electrocatalytic alcohol oxidation reactions (AORs) are processes in which alcohol groups are oxidized to aldehydes, ketones, or carboxylic acids [11, 12, 13, 14, 15, 16, 17, 18]. These processes are important for sustainable energy production, serving as a value‐added anodic process that requires significantly lower energy than the traditional oxidation step in water electrolysis [19, 20, 21]. Furthermore, replacing the sluggish and energy‐intensive oxygen evolution reaction (OER) with the selective oxidation of biomass‐derived alcohols can significantly lower the overall cell voltage required for cathodic hydrogen evolution, while simultaneously generating valuable chemical products at the anode [22, 23, 24, 25]. Thus, a fundamental understanding of catalyst behavior during AORs is essential for improving catalytic performance. Key factors such as the catalyst's morphology, surface structure, and active site distribution can significantly influence its activity. Investigating how morphological features affect catalytic performance can provide valuable insights into the design of more efficient and durable noble metal‐free electrocatalysts.

In general, MOFs have tunable compositions and structures, making them also promising candidates for electrocatalysis in various energy conversion reactions. Their high surface area, well‐defined metal sites, and adjustable coordination environments enable precise control over catalytic activity and selectivity. Previous literature has shown that Ni‐based metal–organic frameworks (MOFs) are stable in alkaline solutions [26, 27, 28], making them suitable for applications such as gas storage/separation [29], heterogeneous catalysis [30], energy materials [31],, etc. In particular, MOF‐based electrocatalysts have shown great potential for reactions such as water splitting and CO_2_ reduction, where the accessibility and electronic configuration of the active metal centers play a decisive role [32, 33]. Thus, understanding the structure–activity relationship under realistic electrochemical conditions remains challenging, since MOFs often undergo dynamic structural and electronic transformations during catalysis. To address this, operando investigation techniques‐especially X‐ray absorption spectroscopy (XAS)‐have become indispensable tools for tracking the evolution of local coordination environments and oxidation states of metal centers under working conditions [34]. Operando XAS provides direct insight into how the active species form, transform, and interact with reactants, thereby revealing the mechanistic origins of catalytic performance. Such studies are particularly valuable for transition‐metal‐based MOFs, where subtle variations in metal‐ligand coordination or morphology can profoundly alter catalytic behavior.

Here, we present a new Ni‐based metal–organic framework (Ni‐MOF) with N‐ and O‐donor linker coordination, which we have chosen as a strategy for alkaline‐medium anodic oxidation reaction. We have used two different synthesis approaches: solvothermal and mechanochemical methods. These synthesis routes yield the same Ni‐based MOF termed as MC‐MOF and ST‐MOF by mechanochemical and nonmechanochemical processes, respectively, which have distinctly different morphologies. A systematic investigation of these materials reveals that the mechanochemically synthesized catalyst exhibits superior activity toward AORs, which can be attributed to its unique morphology and the presence of more accessible and active catalytic sites toward alcohols.

## 2 Results and Discussion

**FIGURE 1:**
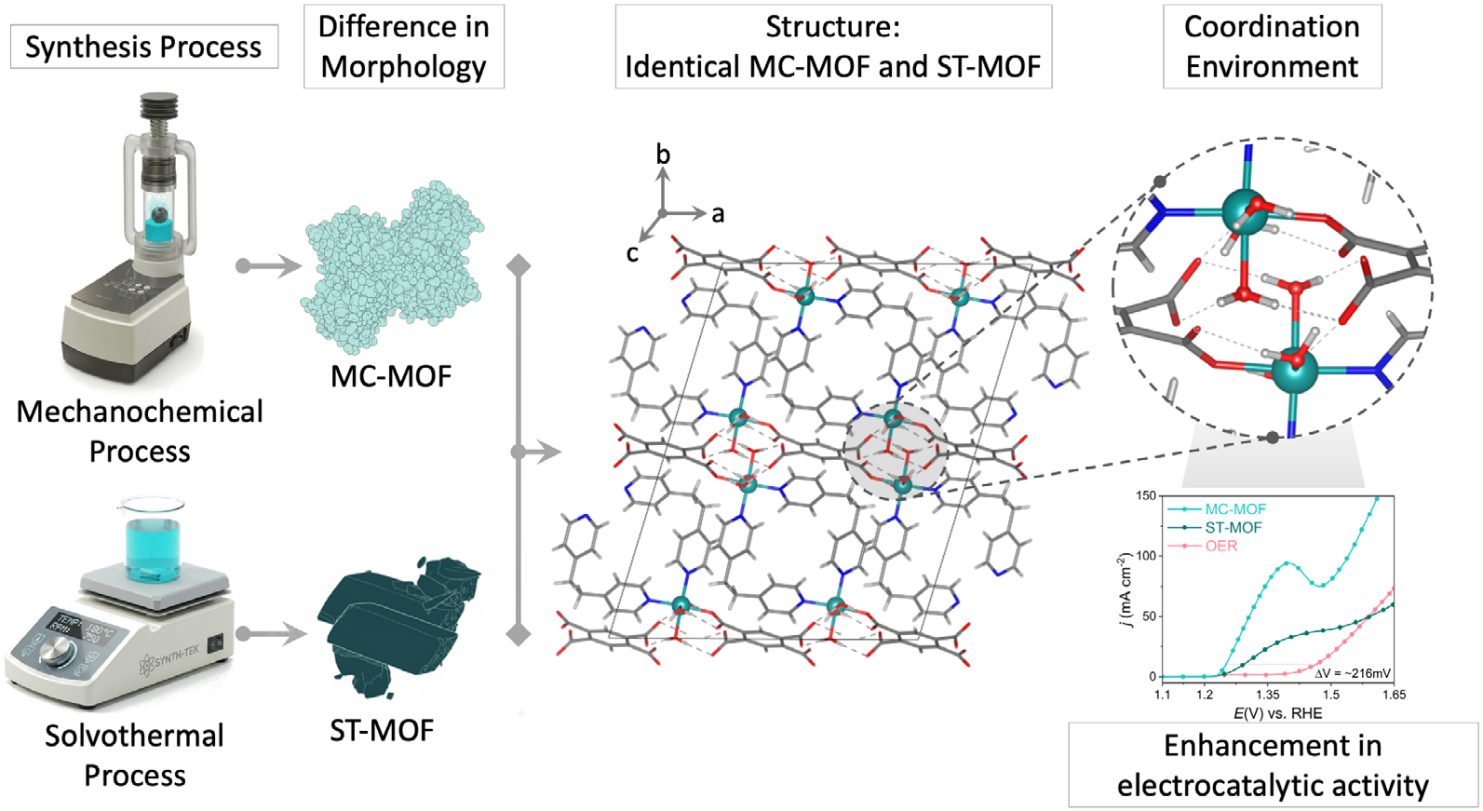
Perspective view of Ni‐based MOF and illustrating the coordination environment Ni‐sites inside the framework. The overall schematic shows two different synthesis methods for the Ni‐based MOF catalyst: the mechanochemical process (MC) and the nonmechanochemical solvothermal route (ST).

**FIGURE 2:**
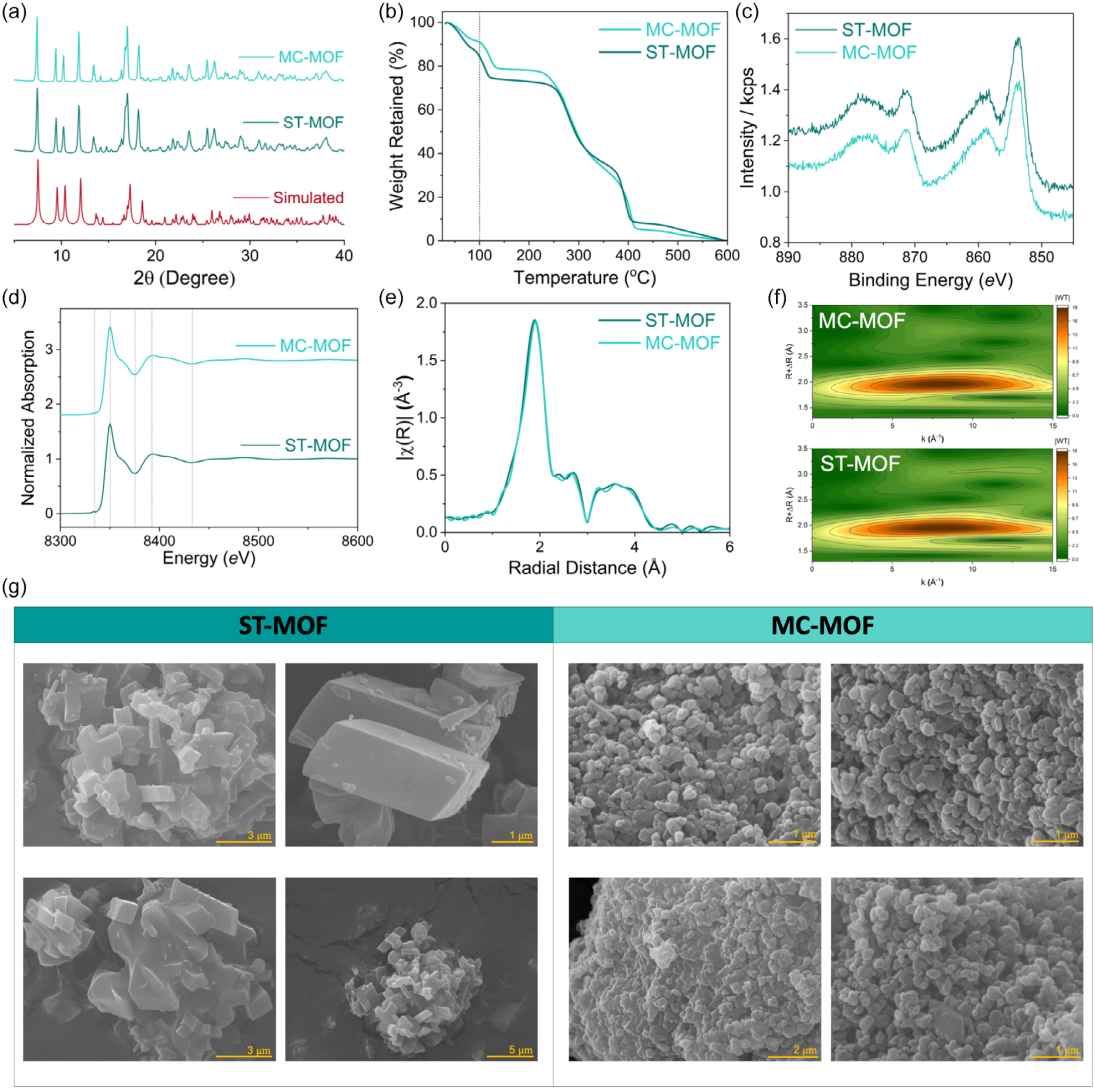
(a) Structural characterizations by PXRD of Ni‐based MOF in both forms ST and MC in comparison with simulated pattern. The intensity has been normalized for each of them, which make intensity axis trivial. (b) TGA of MC‐ and ST‐MOFs. (c) X‐ray photoelectron spectroscopy (XPS) spectra of MC‐ and ST‐MOFs. (d) X‐ray absorption near edge structure (XANES) spectra features electronic transitions from the atomic K‐edge of Ni in MC‐MOF and ST‐MOF. (e) Fourier‐transformed k^2^‐weighted extended X‐ray absorption fine structure (EXAFS) with phase shift depicting local structure environment surrounding Ni‐centers. (f) Wavelet transformation of EXAFS spectra to provide combined resolution in both k‐space and R‐space. (g) SEM images of ST‐MOF and MC‐MOF.

First, a slow diffusion layering method was used to synthesize the Ni‐based MOF at room temperature, resulting in single crystals consisting of mixed ligands, including pyromellitic acid (O‐donor) and 1,2‐bis(4‐pyridyl)ethane (N‐donor). The crystal structure of the MOF was determined using single‐crystal X‐ray diffraction (SCXRD). The crystal structure of the MOF was found to be in the monoclinic C2/c space group and contains octahedral Ni centers coordinated by two pyridine moieties from two different bipyridyl ethane molecules and one carboxylic acid group from pyromellitic acid. The remaining three coordination sites are occupied by water molecules. (see Figure 1) Therefore, Ni‐centers are alternatively coordinated by N‐donor (1,2‐bis(4‐pyridyl)ethane) and O‐donor (pyromellitic acid) linkers forming a 1‐dimensional chain structure. Further, the 1‐dimensional chains are connected via H‐bonding between the coordinated water molecules on the Ni‐center and the carboxylate moiety from the pyromellitic acid from a neighboring chain, overall forming a 2‐dimensional layer structure. The same Ni‐based MOF was synthesized via two additional methods: a mechanochemical approach and a solvothermal method. The resulting MOF products were termed MC‐MOF and ST‐MOF, respectively. Notably, the mechanochemical approach offers significant practical and green‐chemistry advantages, the MC‐MOF requires only 30 min of milling and a minimal 113 μL of solvent, in contrast to the solvothermal approach, which requires 4 h of heating and approximately 40 mL of solvent. The bulk phase purity and structural similarity between MC‐MOF and ST‐MOF were confirmed using powder X‐ray diffraction (PXRD) of the as‐synthesized materials (Figure 2a). The close resemblance between the two fourier‐transform infrared spectroscopy (FTIR) spectra demonstrates that MC‐MOF and ST‐MOF are chemically similar, as they possess the same organic linkers and coordination environment. The strong peaks around 1600–1700 cm^−1^ can be attributed to C=O stretching vibrations from carboxylate groups, while the absorptions near 1000–1300 cm^−1^ are associated with C–O stretching. The broad bands around 3400 cm^−1^ indicate the presence of –OH groups, likely from coordinated or adsorbed water molecules (Figure S2) [35, 36]. Therefore, MC‐MOF and ST‐MOF exhibit similar structural features, confirming their identical PXRD and FTIR data. However, the SEM images reveal that mechanochemical synthesis (MC‐MOF) yields significantly smaller and agglomerated particles compared to the larger, well‐defined crystals of ST‐MOF (Figure 2g). Thermogravimetric analysis (TGA) indicates that both MOFs exhibit comparable thermal stability up to ∼250°C (Figure 2b). However, ST‐MOF reveals a higher weight loss below ∼250°C compared to MC‐MOF. This suggests that the mechanochemically synthesized MC‐MOF with a minimal solvent used (∼113 μL) either contains fewer coordinated water molecules at the Ni sites or has fewer guest solvent molecules entrapped within the pores overall. Due to the solvent‐rich environment of the solvothermal synthesis, it is likely that ST‐MOF incorporates more guest molecules, contributing to its higher overall weight loss. It is important to note that both MOFs were thoroughly washed with water after synthesis to remove by‐products such as acetic acid from the acetate precursor salts and to purify the MOF materials.

X‐ray photoelectron spectroscopy (XPS) spectra of both the MC‐MOF and the ST‐MOF show characteristic Ni^2+^ peaks in the Ni 2p region. The two main peaks are at ∼855.8 eV and Ni ∼873.4 eV (Figure 2c). These binding energies indicate that the oxidation state of Ni is +2 and the electronic environment around the Ni centers remains the same in both the MC‐ and ST‐MOFs. The presence of satellite peaks in the range of ∼861–865 eV and ∼879–883 eV further supports the assignment to Ni^2+^ [37]. The similar features of the spectra of both samples confirm that the Ni centers in both MC‐MOF and ST‐MOF are electronically equivalent (Figure S5 and S8).

To elucidate the local‐structural and electronic properties of the synthesized MOF materials, we employed XAS. The X‐ray absorption near edge structure (XANES) spectra (Figure 2d) for both MC‐MOF and ST‐MOF exhibit the Ni K‐edge at 8345.6 eV, unequivocally confirming a uniform + 2 oxidation state for the Ni centers [38]. These findings, corroborated by XPS and XANES, established that both synthesis methods yield electronically analogous materials, which is crucial for comparing any electrocatalysis performance in the next step of our studies. Furthermore, the local coordination environment around Ni centers is probed by extended X‐ray absorption fine structure (EXAFS), which is identical for both MC‐ and ST‐MOFs (Figure 2e). The spectra are dominated by a peak at 1.9 Å, corresponding to the Ni‐O bond distance, and the congruence in peak position and magnitude signifies an identical coordination number. This high degree of structural fidelity implies that both frameworks are well‐formed without significant defects or missing linkers around the Ni‐local environment. Therefore, the greater weight loss observed in the TGA data for the ST‐MOF (Figure 2b) can be confidently attributed to a higher amount of solvent occluded within its pores, rather than any difference in framework integrity or coordination number of Ni centers. To quantitatively analyze the local coordination environment, the EXAFS spectra of both MC‐ and ST‐MOFs were fitted using a structural model derived from SCXRD data. In both materials, the first coordination shell consists of six neighboring atoms, consistent with the SCXRD‐derived structural model. The fitting results are presented in Figure S9 and summarized in Table S3. As a complementary technique, Wavelet Transformation (WT) of the EXAFS spectra was employed to effectively resolve and visualize the backscattering contributions from specific neighboring atoms [39]. The WT‐EXAFS plots for both materials are nearly identical, exhibiting a primary intensity maximum at the same position. This dominant feature is attributed to the backscattering signal of neighboring oxygen and nitrogen atoms in the first coordination shell (Figure 2f). This finding further supports the conclusion that both synthesis methods produce materials with indistinguishable local structures around the metal centers.

**FIGURE 3:**
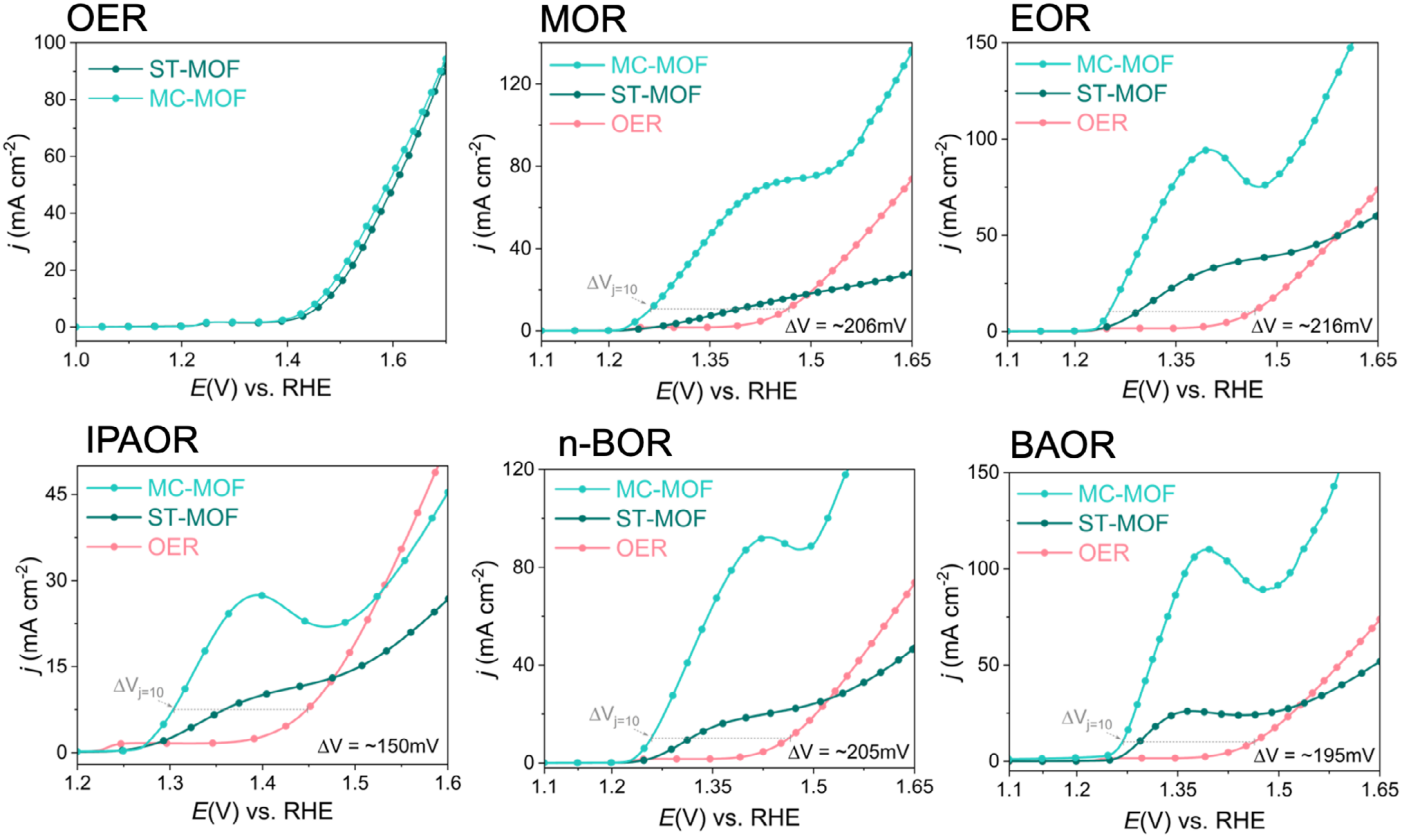
Comparison between MC‐MOF and ST‐MOF as electrocatalysts for different oxidation reactions such as the OER, MOR, EOR, IPAOR, n‐butyl alcohol oxidation reaction (n‐BOR), and BAOR. All LSV curves were recorded at a scan rate of 5 mV s^−1^. The electrolyte composition consisted of 1.0 M NaOH for the OER, and 1.0 M NaOH containing 0.1 M of the respective alcohol for the AORs. Data presentation: The displayed LSV curves are representative initial scans selected from three independent electrode preparations. This format was chosen to maintain visual clarity while reproducible and precluding the need for error bars or probability value testing.

The electrocatalytic performance of MC‐MOF and ST‐MOF was evaluated in terms of the OER in 1 M NaOH and for various AORs in 0.1 M alcohol solutions containing 1 M NaOH. Linear sweep voltammetry (LSV) showed that both MOFs exhibited comparable activity toward the OER during the initial measurements. However, MC‐MOF consistently demonstrated significantly higher activity for all AORs. We attribute this difference to the nature of the reactants and how they interact with the Ni‐active centers. For OER, water or hydroxide molecules are abundant in the electrolyte and readily available at Ni sites, even in the pristine form. This results in similar catalytic performance for both MC‐MOF and ST‐MOF. However, in AORs, the alcohol molecules must first adsorb onto the available surface Ni sites to undergo oxidation. Since MC‐MOF has a smaller particle size and thus a higher surface area, it likely exposes more accessible Ni‐active centers, enabling more efficient alcohol adsorption and oxidation compared to ST‐MOF. To quantitatively verify that this enhancement is driven by active site density rather than a change in intrinsic site activity, the electrochemically active surface area (ECSA) was evaluated by measuring the electrochemical double‐layer capacitance (*C*_*dl*_) in the non‐Faradaic region (Figure S16). The MC‐MOF exhibited a *C*_*dl*_ of 0.093 mF cm^−2^, which is approximately 4.4 times higher than that of the ST‐MOF (0.021 mF cm^−2^). Because our spectroscopic analyses confirmed that the local coordination and electronic state of the Ni centers are completely identical in both materials, this significant increase in ECSA definitively proves that the superior geometric catalytic performance of the MC‐MOF stems directly from its highly agglomerated, nanoscale morphology, exposing a substantially greater number of accessible active sites to the electrolyte. To achieve a geometric current density of 10 mA cm^−2^, the MC‐MOF required a substantially lower overpotential for AORs compared to the OER, with reductions of overpotential approximately ∼206 mV for methanol oxidation reaction (MOR), ∼216 mV for ethanol oxidation raection (EOR), ∼150 mV for isopropyl alcohol oxidation reaction (IPAOR), ∼205 mV for n‐butyl alcohol (n‐BOR), and ∼195 mV for benzyl alcohol oxidation reaction (BAOR) (Figure 3). Additionally, the chronopotentiometry technique has been used to validate the requirement of the lower overpotential MC‐MOF at 10 mA cm^−2^ for all the different types of alcohols (Figure S14). Furthermore, due to the bulkiness of the organic substrates, the AOR operates under mixed kinetic‐diffusion control, which precludes the extraction of standard Tafel slopes without significant mass‐transport deconvolution. To further evaluate the long‐term durability of the catalyst under continuous anodic operation, we conducted an extended 24 h chronopotentiometry test for the EOR. The MC‐MOF demonstrated excellent functional stability, sustaining the required current density without a significant increase in overpotential over the entire 24 h period (Figure S17). It is important to note that while MOFs can undergo dynamic structural transformations during prolonged exposure to alkaline electrocatalytic conditions, the sustained functional stability observed here indicates that the MC‐MOF acts as a robust precursor. Its unique initial coordination environment guides a controlled evolution into a highly reactive and stable active phase that consistently drives the oxidation reaction.

To identify the products, alcohol oxidation was performed using chronopotentiometry for 4 h long, and the resulting electrolyte was analyzed by proton nuclear magnetic resonance (^1^H‐NMR). The NMR analysis after EOR confirmed the formation of acetate in the alkaline medium, evidenced by a new singlet proton (^1^H‐NMR) peak appearing at δ ∼1.37 ppm in the pristine alkaline electrolyte for the methyl group of the acetate salt (Figure S10). Secondary alcohols like isopropanol can be oxidized to ketones, making this a 2‐electron oxidation process without any further oxidation like primary alcohols. So, the electrolyte after IPAOR was analyzed by ^1^H‐NMR and reveals a new singlet proton at δ ∼1.7 ppm, by the identical methyl groups of acetone (Figure S11). Following the n‐Butanol Oxidation Reaction, ^1^H‐NMR analysis of the electrolyte confirms the formation of the butyrate anion as the primary product. The ^1^H‐NMR spectrum shows the following characteristic signals: δ ∼0.89 ppm (Triplet, 3H): This signal corresponds to the terminal methyl (−CH_3_) protons. δ 1.58 ppm (Sextet, 2H): This signal is assigned to the protons of the middle methylene group (−CH2−). δ 2.18 ppm (Triplet, 2H): This peak represents the protons of the methylene group alpha (α) to the carboxylate group (−CH2−COO−) (Figure S12). In the case of BAOR, the NMR results indicate the oxidation of benzyl alcohol to benzoic acid. The final product is identified by the two multiples in the aromatic region. The signals at δ ∼7.3‐7.4 ppm (2H) and δ ∼6.9‐7.0 ppm (3H) are characteristic of the aromatic protons on a benzoate ring. The two ortho protons shift further downfield (δ ∼7.3‐7.4 ppm) due to the strong electron‐withdrawing effect of the carboxylate group. Meanwhile, the meta and para protons appear slightly more upfield. Therefore, the product resulting from this benzyl alcohol oxidation reaction is benzoate, which is likely to exist as a benzoate salt in the final sample (see Figure S13).

**FIGURE 4:**
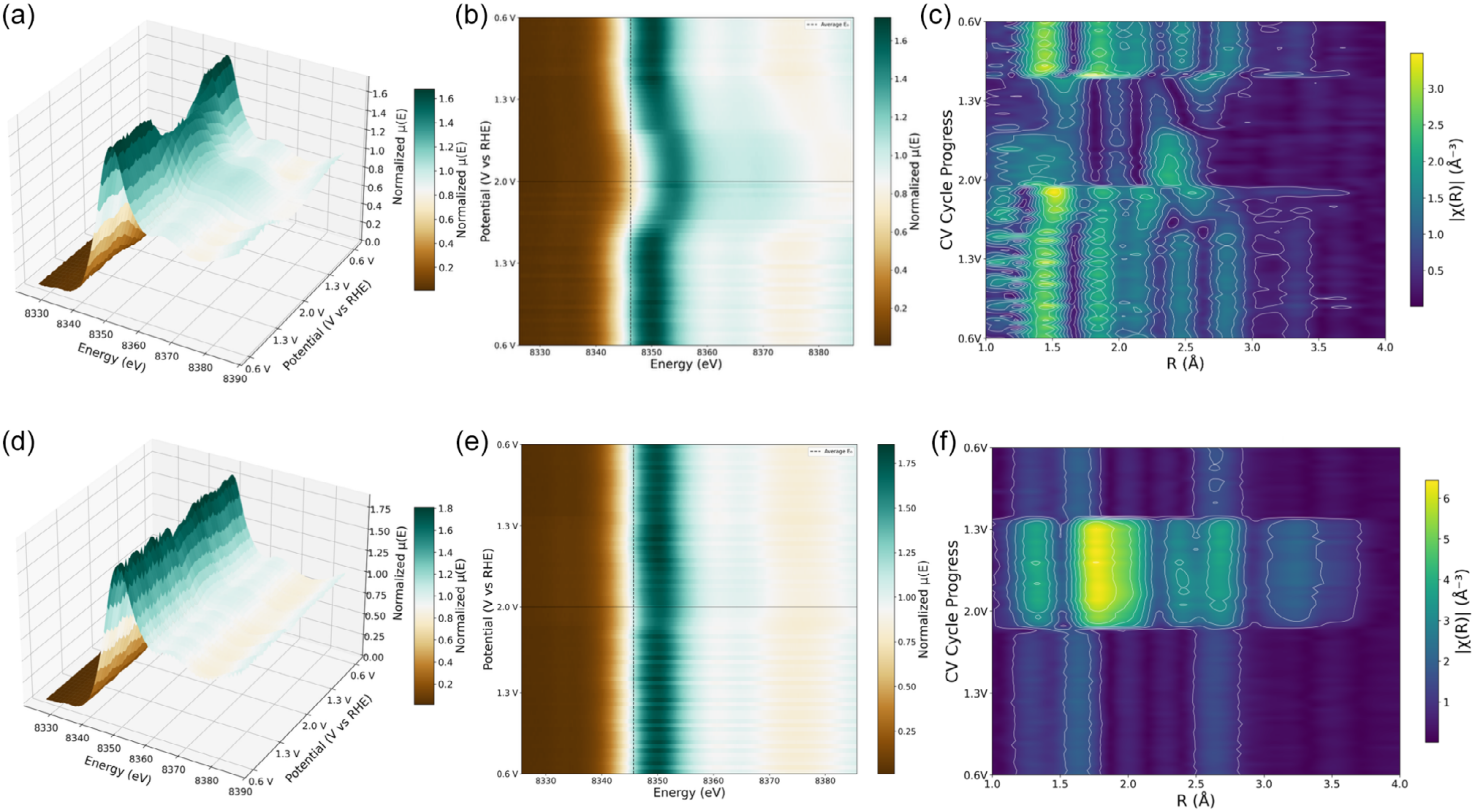
Operando Quick‐XAS of Ni K‐edge during OER (a)–(c) and EOR (d)–(f). (a,d) 3D surface representation of the XANES evolution as a function of applied potential. (b,e) Corresponding contour plots of normalized absorption versus energy and potential. (c,f) Potential‐dependent Fourier‐transformed EXAFS spectra, highlighting structural changes in the local Ni environment under reaction conditions.

In order to provide insights into real‐time structural and electronic transformations during the OER and EOR process on the MC‐MOF catalyst, operando quick‐XAS [40, 41] was carried out using a custom‐made electrochemical cell (Figure S16). As the applied potential increases, a positive shift of the Ni‐K edge is observed, and is directly correlated to the oxidation of the Ni‐centers. Figure 4a shows the whole XANES spectra with respect to the applied potential, and Figure 4b shows the 2‐D plots of the edge‐position (in eV) with respect to potential. A higher potential provides the driving force for the oxidation of the pristine Ni^+2^ in the MOF to a higher valence state +3 or even +4. The High‐valent Ni‐oxygenated species have been observed in XAS during OER electrocatalysis in alkaline medium as the reactant of the rate‐determining step [42, 43, 44]. This has previously been proven when investigating the OER mechanism in previous literature reports [42, 45]. Figure 4c shows the resulting magnitude of the chi(k) versus R, which is proportional to the coordination environment around Ni throughout the applied potential range. Therefore, Ni‐centers in the MC‐MOF undergo the formation of high‐valent Ni‐Oxygenated species in alkaline medium during OER, and the heatmap of EXAFS shows the evolution of local structure, peaks at ∼1.5–1.6 Å (without phase‐shift). EXAFS amplitude was reduced upon the formation of higher‐valent Ni‐oxygenated (+3/+4) species, which results in a lower amplitude because less atoms contribute to the same radial distance at the higher oxidative potential (∼2 V vs. RHE). The Ni K‐edge during the EOR shows a very small change in edge position, indicating a different reaction pathway in the presence of ethanol molecules in the alkaline medium and preference toward EOR over OER on Ni‐centers (Figure 4d,e). The strong adsorption of the alcohol molecules on the Ni‐centers effectively suppresses the accumulation of the high‐valent active oxygenated species on the Ni‐centers, even in an alkaline medium, and follows the EOR path. Additionally, the MOF structure exhibits a feasible arrangement of strong interactions via hydrogen bonding between the water molecules and the carboxylate group or products after alcohol oxidation on the Ni‐centers (Figure 1). This hindrance of product desorption suggests that these adsorbed species act as the reactants in the rate‐determining step. Consequently, their presence significantly influences the EXAFS spectra [42]. Therefore, during EOR with potential, the EXAFS intensity strengthens as the reaction carries on for more time and forms a protective layer of carboxylate‐based products on the Ni‐centers, while staying at the +2 oxidation states (Figure 4e,f). Based on our operando XAS observations and NMR product analysis, we propose a comprehensive mechanism for alcohol oxidation on the MC‐MOF, detailed in the Supporting Information (Note S4 and Figure S20). The catalytic cycle initiates with alcohol adsorption and deprotonation to form a Ni^2+^‐alkoxy intermediate. Subsequent proton electron transfer steps generate highly reactive, transient Ni^3+^ species. These transient high‐valent centers rapidly drive the dehydrogenation of the substrate‐yielding ketones via a 2‐electron pathway for secondary alcohols, or carboxylates via a complete 4‐electron pathway for primary alcohols, before immediately returning to the stable Ni^2+^ resting state. This mechanistic model perfectly elucidates our operando XAS results, because the high‐valent Ni^3+^ states are highly transient and rapidly consumed by the alcohol substrate, they do not accumulate. Consequently, Ni^2+^ dominates the absorption spectra as the catalytic resting state during AOR, in contrast to the stable high‐valent Ni‐Oxygenated species that accumulate during OER. This comprehensive analysis of operando XAS data shows the divergent behavior of the same Ni‐based MC‐MOF catalyst in the presence of small organic alcohol molecules in an alkaline medium, and its selectivity toward EOR over OER. This is achieved through fundamentally different mechanisms depending on the preference of the absorbed reactant molecule.

## 3 Conclusions

In summary, we demonstrated that mechanochemical synthesis produces a Ni‐based MOF that exhibits distinct morphological advantages, significantly enhancing its performance as a value‐added anodic process for alcohol oxidation compared to the solvothermally synthesized analog. Crucially, comprehensive spectroscopic analyses (XPS and XAS) confirmed that both synthesis routes yield an identical local coordination environment and electronic state at the Ni active centers. As a result, the enhanced activity is strictly geometrically driven, the mechanochemically induced morphology yielded a roughly 4.4‐fold higher ECSA, driving the selective oxidation of various alcohols to valuable products while maintaining excellent functional stability over 24 h of continuous operation. Operando XAS revealed different catalytic pathways under OER and AOR conditions, showing that reactant‐specific interactions govern Ni‐center activity by suppressing high‐valent Ni‐oxygenated species. The findings emphasize the potential of mechanochemical strategies for designing efficient, sustainable, and morphology‐tuned MOF‐based electrocatalysts.

## Supporting Information

Additional supporting information can be found online in the Supporting Information section.

## Conflicts of Interest

The authors declare no conflicts of interest.

## Supporting information

Supplementary Material

## Data Availability

The data that support the findings of this study are available from the corresponding author upon reasonable request.
