# Supplementary material for: Operando Investigation of Mechanochemically Synthesized Ni‐Based Metal–Organic Frameworks for Electrocatalytic Alcohol Oxidation
Source: ChemSusChem. 2026 Apr 21;19(8):e202502581. doi: 10.1002/cssc.202502581 (PMC13099272; doi:10.1002/cssc.202502581)
Supplement: Supplementary file 1 — Supplementary Material [file CSSC-19-e202502581-s001.pdf]

Supporting Information

Operando Investigation of Mechanochemically Synthesized Ni-based Metal Organic Frameworks for Electrocatalytic Alcohol Oxidation

Arkendu Roy,*[a,b] Xavier Vaneberck,[a] Celia Ganado Rodriguez,[a] Semen Butyrin,[a] Dominik Al-Sabbagh,[a] Chayanika Das,[a] Klas Meyer,[a] Ines Feldmann,[a] Jörg Radnik,[a] Ana Guilherme Buzanich,*[a] Franziska Emmerling,*[a,b] Biswajit Bhattacharya*[a]

[a] Federal Institute of Materials Research and Testing (BAM)

Address: Richard-Willstätter-Str 11, 12489 Berlin, Germany

[b] Humboldt University, Germany

Address: Rudower Ch 25, 12489 Berlin, Germany

* Corresponding Author

Email: arkendu.roy@bam.de; ana.buzanich@bam.de; franziska.emmerling@bam.de; biswajit.bhattacharya@bam.de

Materials:

All chemicals were used as received without further purification. 1,2-bis(4-pyridyl)ethane, tetrasodium pyromellate, pyromellitic acid, and nickel(II) acetate tetrahydrate were employed as the organic linkers and metal precursor for Ni-MOF synthesis. Methanol (MeOH), ethanol (EtOH), and deionized water (H_2O) were used as solvents for crystal growth, solvent-assisted synthesis, and washing procedures.

Synthesis procedure:

Crystal Growth of Ni-MOF, $[Ni(BPE)(PM)_{0.5}(H_2O)_3]\cdot G$:

A methanolic solution (20 mL) of 1,2-bis(4-pyridyl)ethane (BPE) (1 mmol, 0.184 g) was mixed with an aqueous solution (20 mL) of tetrasodium pyromellate (Na_4-PM) (1 mmol, 0.342 g), and the resulting solution was stirred for 15 min to mix well. Nickel(II)-acetate tetrahydrate (1 mmol, 0.249 g) was dissolved in 20 mL of water in a separate beaker. Six milliliters of this mixed ligand solution was slowly and carefully layered above 3 mL of metal solution using 2 mL of buffer (1:1 of water and MeOH) in a glass tube. After 1-week, green single crystals suitable for single crystal X-ray diffraction were obtained on the inner wall of the tube.

Solvent based synthesis of Ni-MOF:

For the bulk synthesis of Ni-MOF, aqueous solution of corresponding metal was mixed with above-mentioned mixed ligand solution. The above solution was stirred for 4 hours at 65 ^{o}C temperature and then the precipitate was filtered and air-dried, and phase purity was confirmed by IR spectra, thermal analysis, and powder X-ray diffraction for further characterizations.

Mechanochemical Synthesis:

1,2-bis(4-pyridyl) ethane (BPE) (1 mmol) and pyromellitic acid (PA) (1 mmol) was added to a oval-shaped mechanochemical jar made of stainless steel with two 10mm stainless steel balls. Next, the jar was charged with Ni(II)-acetate tetrahydrate (1 mmol) and followed by pre-mixing with a spatula and adding 113 μL of water and ethanol mixture (1:1). The mixture was milled for 30 mins at 50 Hz using a Fritsch Pulverisette 23 for vertical milling. Next, the powder was collected and washed with water and methanol thoroughly to remove the acetic acid (side product). Finally, the power was collected and air-dried at room temperature.

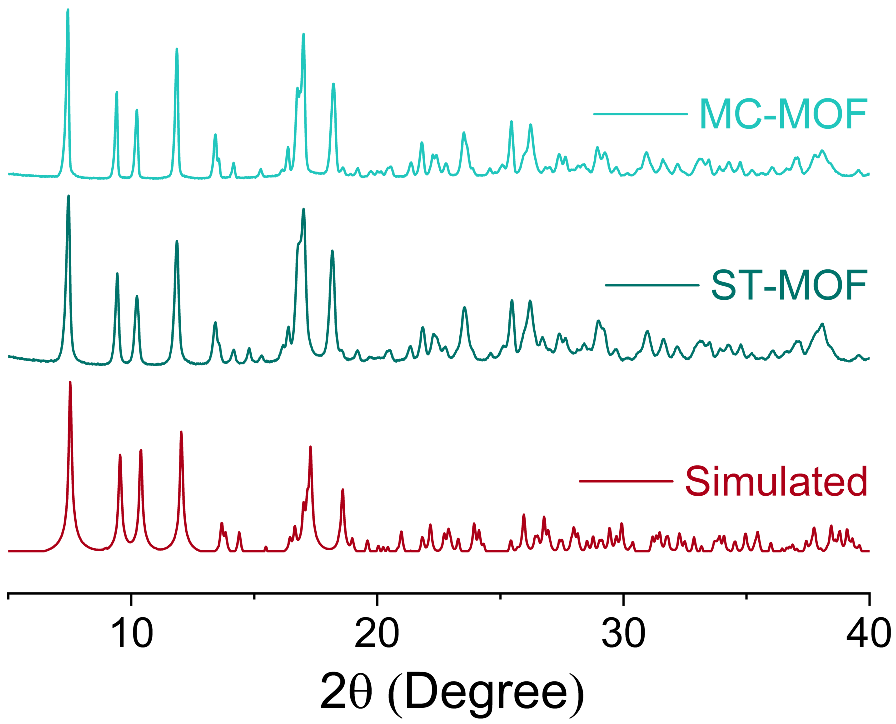

Figure S1. PXRD pattern of MC-MOF and ST-MOF showing crystallinity and phase purity by comparing with the simulated pattern.

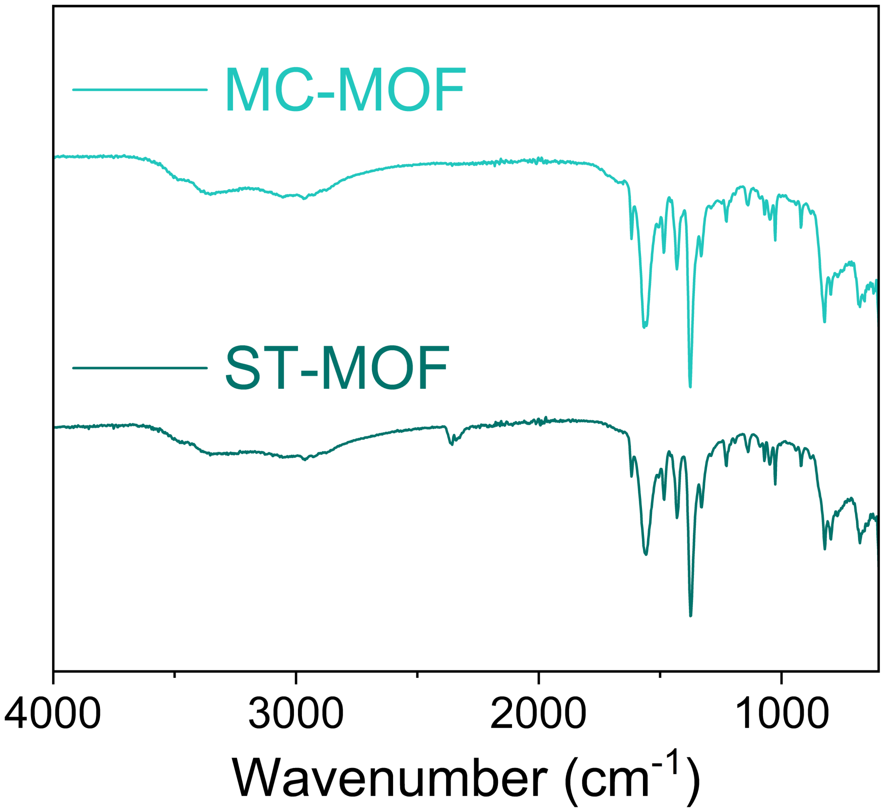

Figure S2. The FTIR spectra of both MC-MOF and ST-MOF exhibit characteristic absorption bands in similar regions, confirming the presence of comparable functional groups.

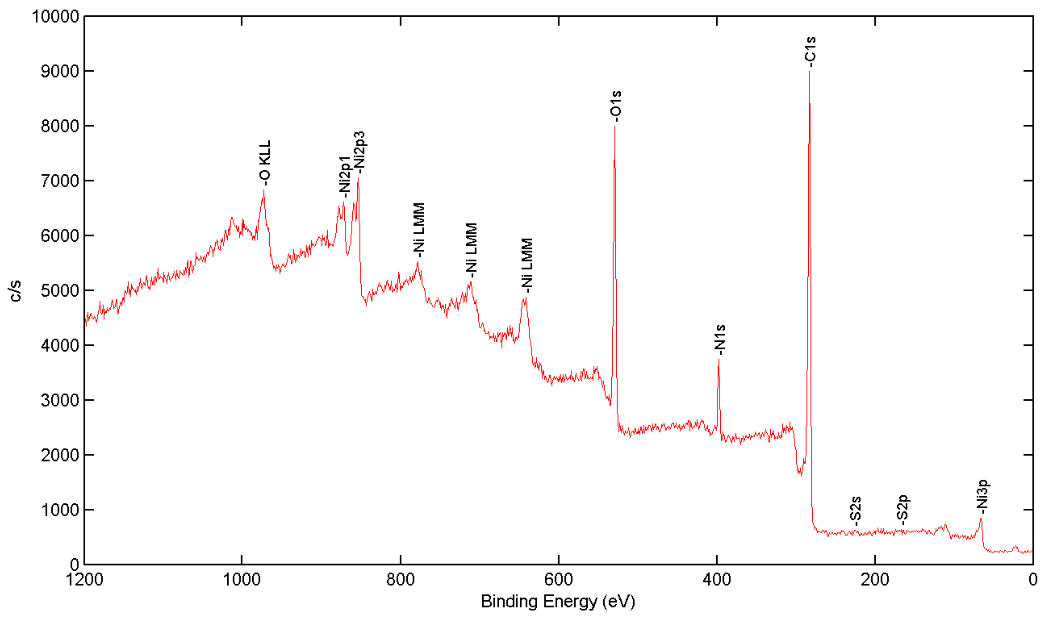

Figure S3. XPS survey Spectrum of ST-MOF, showing the characteristic peaks of Ni, O, C, N.

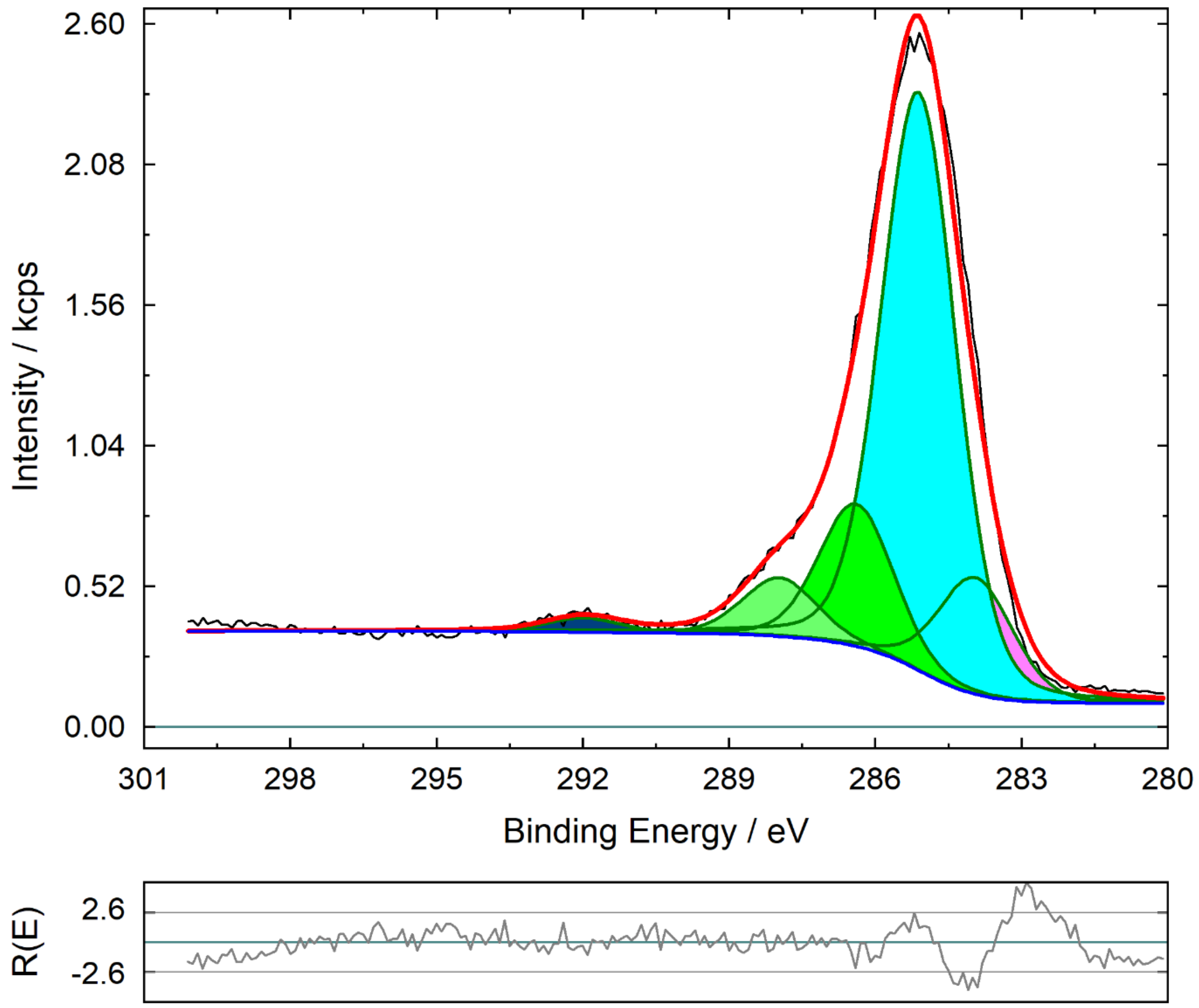

Peak name C1s	Peak height/ cps	Lorentzian	Position/ eV	FWHM/ eV	abs. Area/ cps·eV	rel. Area/ %
C (sp2)	431.319	0.2	284.5496	1.8	883.15	12.75
C (sp3), C-N	2140.4	0.2	285.7169	1.8	4401	63.53
C-O	526.515	0.2	286.9959	1.8	1085	15.67
C=O	216.419	0.2	288.5698	1.8	446.85	6.45
pi-pi*	53.713	0.2	292.5786	1.8	110.92	1.6

Figure S4. High resolution XPS spectra of C of ST-MOF.

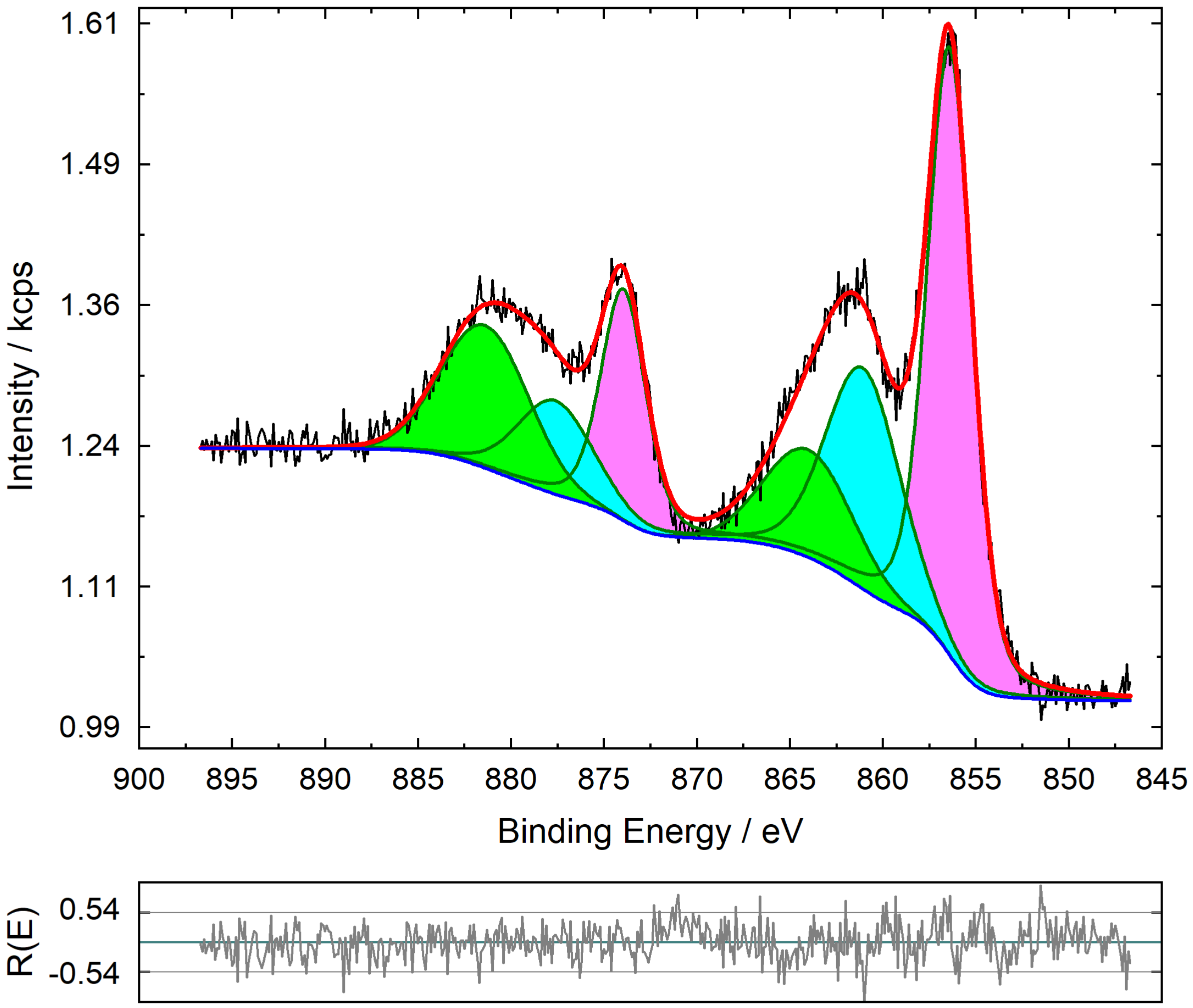

Doublet name Ni2p	Peak height/ cps	Lorentzian	Position/ eV	FWHM/ eV	abs. Area/ cps·eV	rel. Area/ %
Ni (II)	535.369<br>202.989	0.28376<br>0.28376	856.4137<br>873.9588	2.84821<br>2.84821	1803<br>689	34.16<br>13.05
Ni (II) sat.	194.318<br>81.692	0.18968<br>0.18968	861.0803<br>877.6287	4.71426<br>4.71426	1043<br>440	19.76<br>8.33
Ni (II) sat.	94.81<br>125.566	0<br>0	864.0164<br>881.3943	5.55677<br>5.55677	560.82<br>742.75	10.62<br>14.07

Figure S5. High resolution spectra of Ni of ST-MOF.

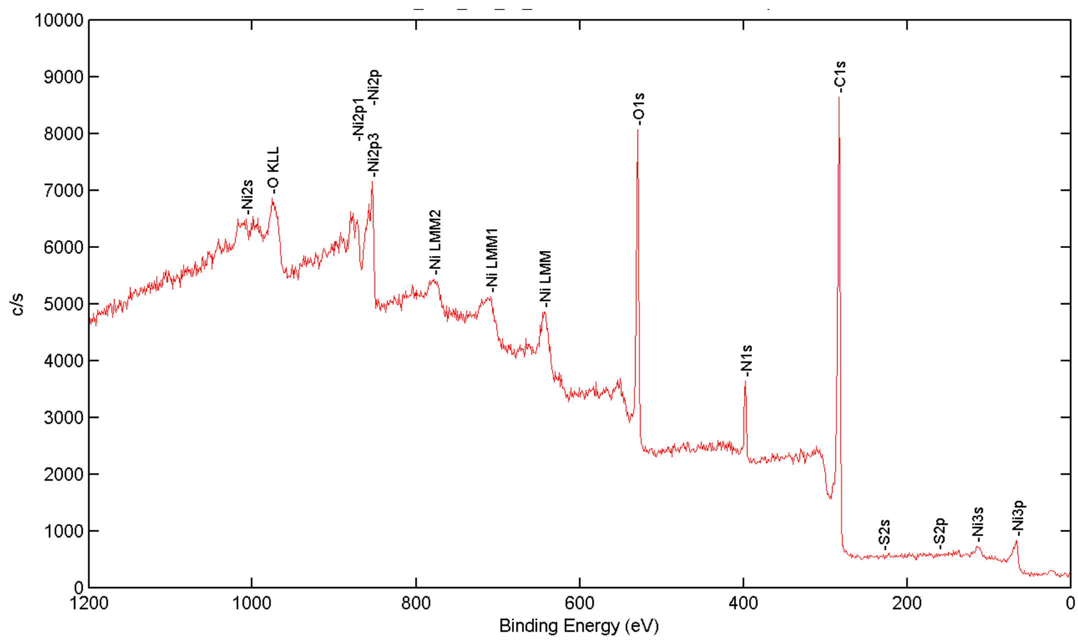

Figure S6. Survey Spectra of MC-MOF, showing the characteristic peaks of Ni, O, C, N.

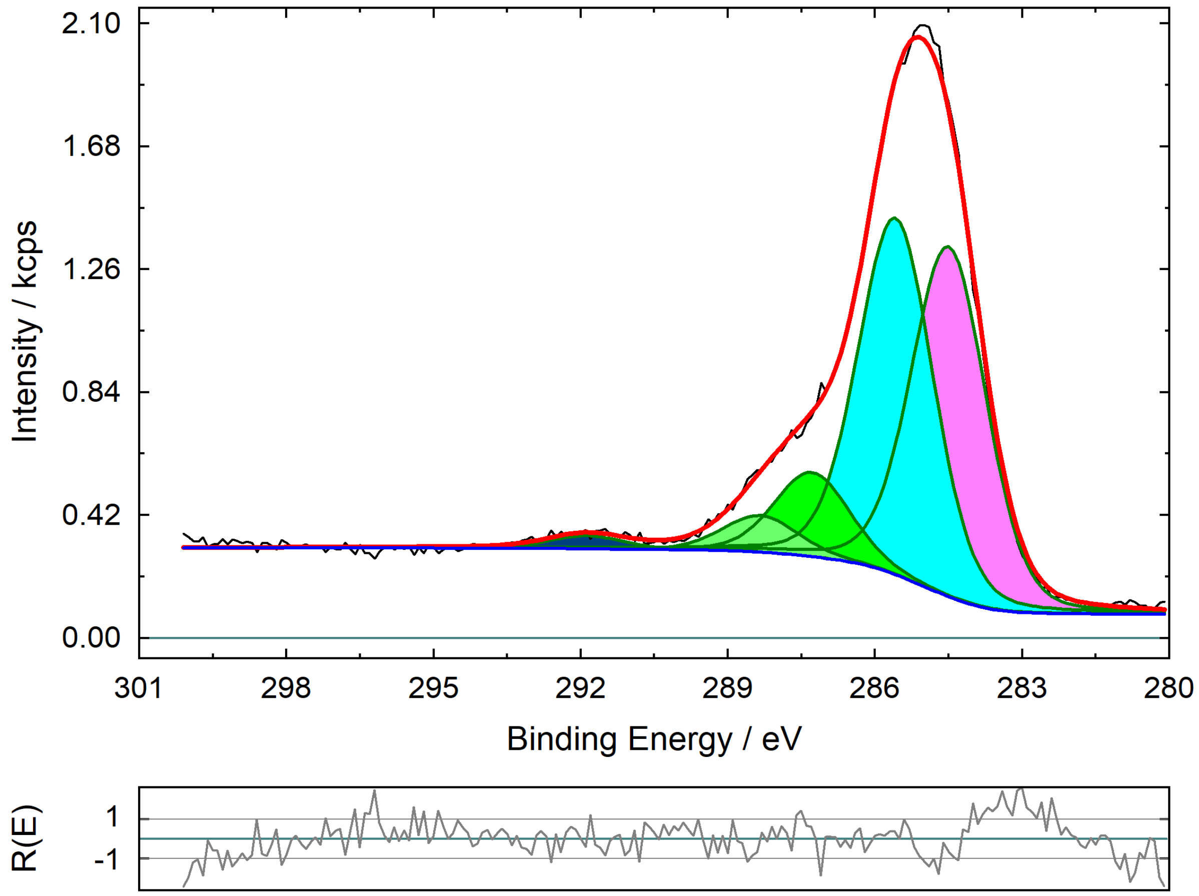

Peak name C1s	Peak height/ cps	Lorentzian	Position/ eV	FWHM/ eV	abs. Area/ cps·eV	rel. Area/ %
C (sp2)	1197.3	0.2	284.4987	1.8	2457	41.56
C (sp3), C-N?	1219	0.2	285.5821	1.8	2509	42.44
C-OH	288.04	0.2	287.288	1.8	594.38	10.05
C=O	125.039	0.2	288.345	1.8	258.23	4.37
pi-pi*	45.066	0.2	291.8859	1.8	93.07	1.57

Figure S7. High resolution XPS spectra of C of MC-MOF.

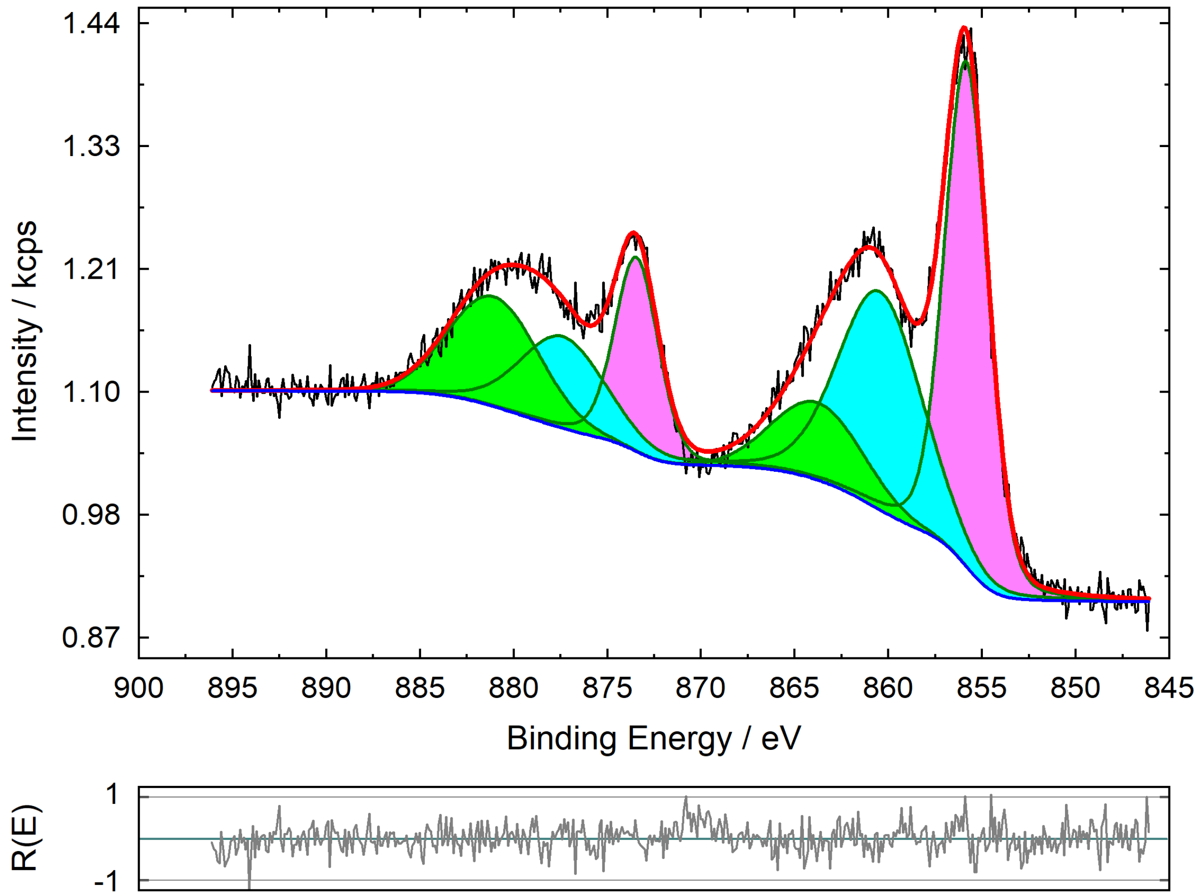

Doublet name Ni2p	Peak height/ cps	Lorentzian	Position/ eV	FWHM/ eV	abs. Area/ cps·eV	rel. Area/ %
Ni (II)	468.428<br>179.718	0.18059<br>0.18059	855.8393<br>873.4468	2.7327<br>2.7327	1460<br>563	30.61<br>11.8
Ni (II) sat.	202.062<br>84.288	0.16495<br>0.16495	860.4382<br>877.3915	5.26519<br>5.26519	1199<br>501	25.14<br>10.51
Ni (II) sat.	71.699<br>101.835	0<br>0	863.7907<br>881.0612	5.66194<br>5.66194	432.14<br>613.78	9.06<br>12.87

Figure S8. High resolution XPS spectra of Ni of MC-MOF.

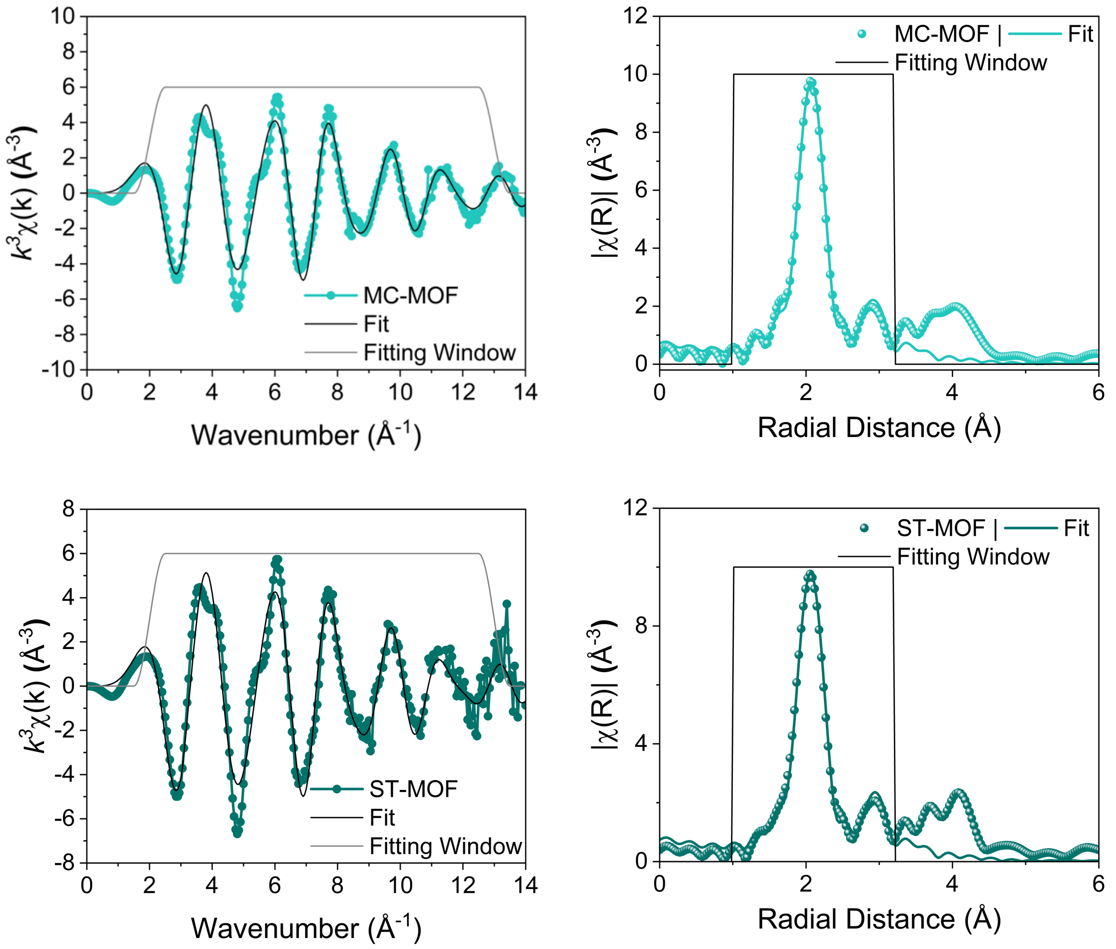

Figure S9. EXAFS fitting results in k-space (left) and R-space (right) for MC-MOF (top) and ST-MOF (bottom) samples and were collected at the Ni K-edge.

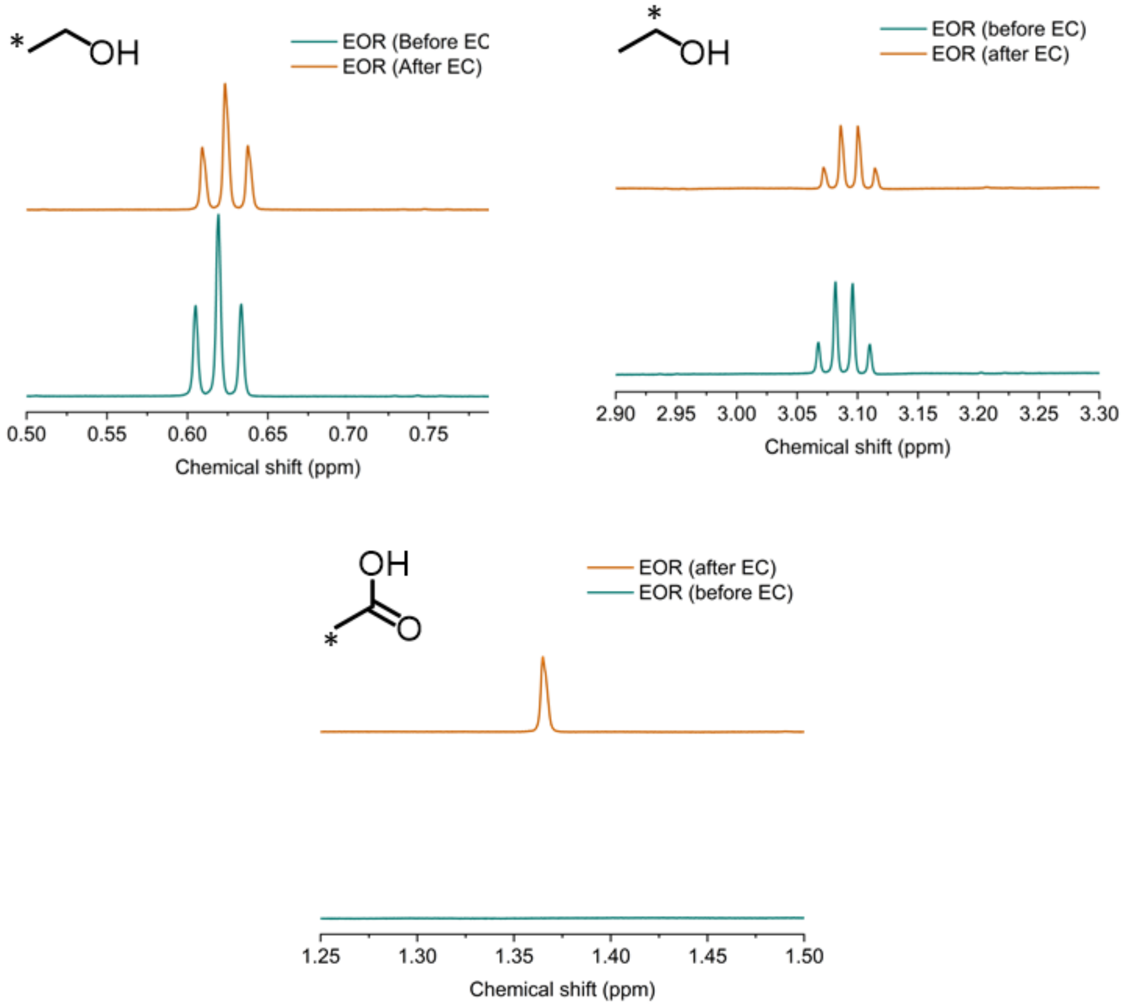

Figure S10. ^{1}H-NMR spectra of ethanol oxidation reaction (EOR) before and after electrochemical (EC) treatment. Signals corresponding to ethanol (CH_3 and CH_2 groups) decrease after EC, while the appearance of new peaks indicates the formation of acetic acid as the main oxidation product.

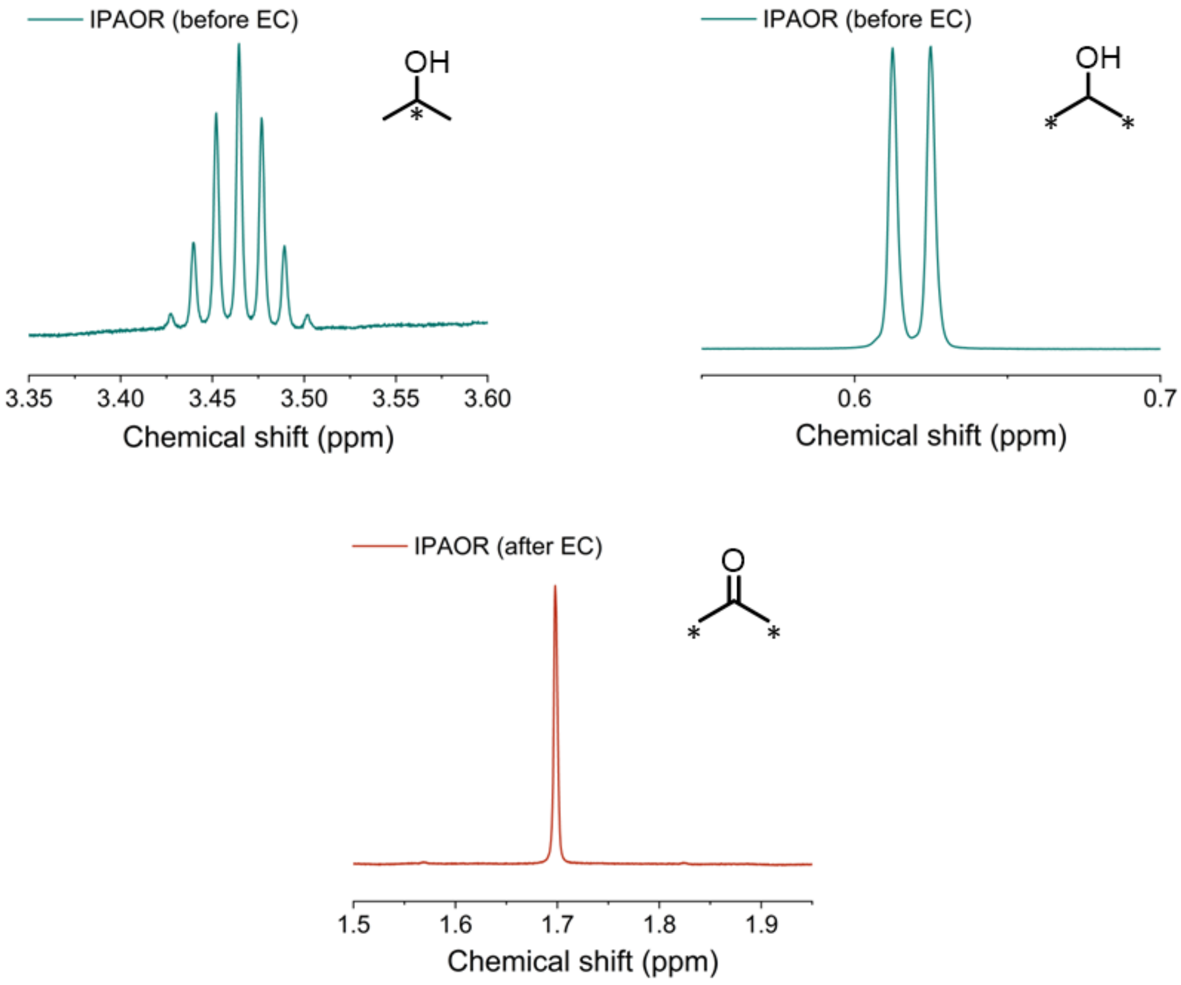

Figure S11. ^{1}H-NMR spectra of isopropanol oxidation reaction (IPAOR) before and after electrochemical (EC) treatment. Characteristic signals of isopropanol (–CH and –CH_3 groups) diminish after EC, while a new peak corresponding to acetone appears, confirming the oxidation of isopropanol to acetone.

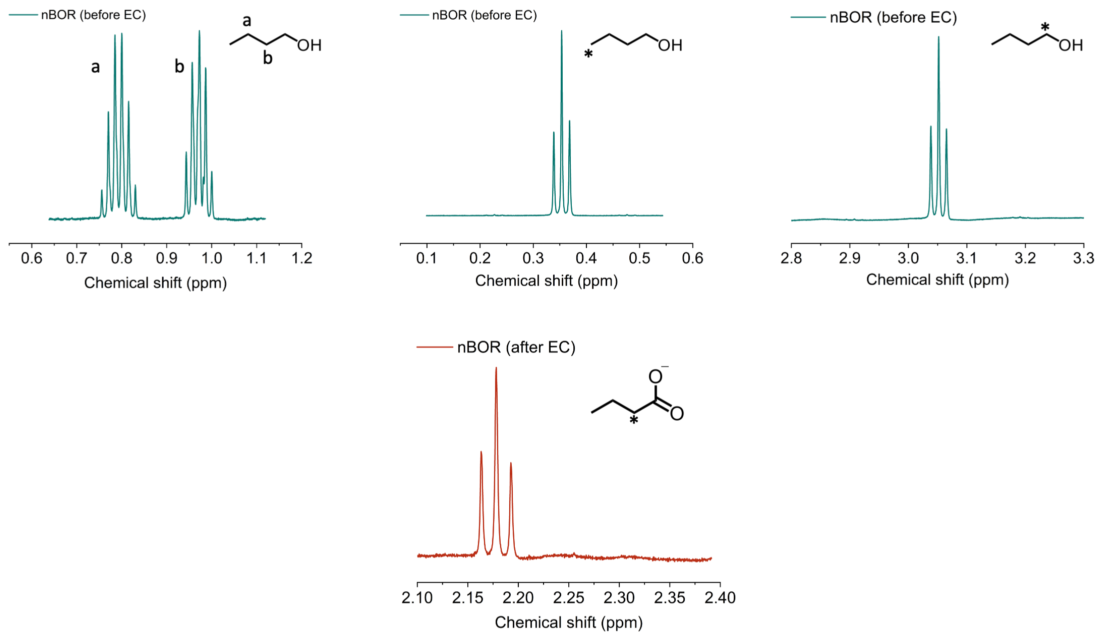

Figure S12. ^{1}H-NMR spectra showing the oxidation of n-butanol (nBOR) before and after electrochemical (EC) treatment. After EC treatment, the emergence of new peaks confirm the formation of butyrate as the oxidation product.

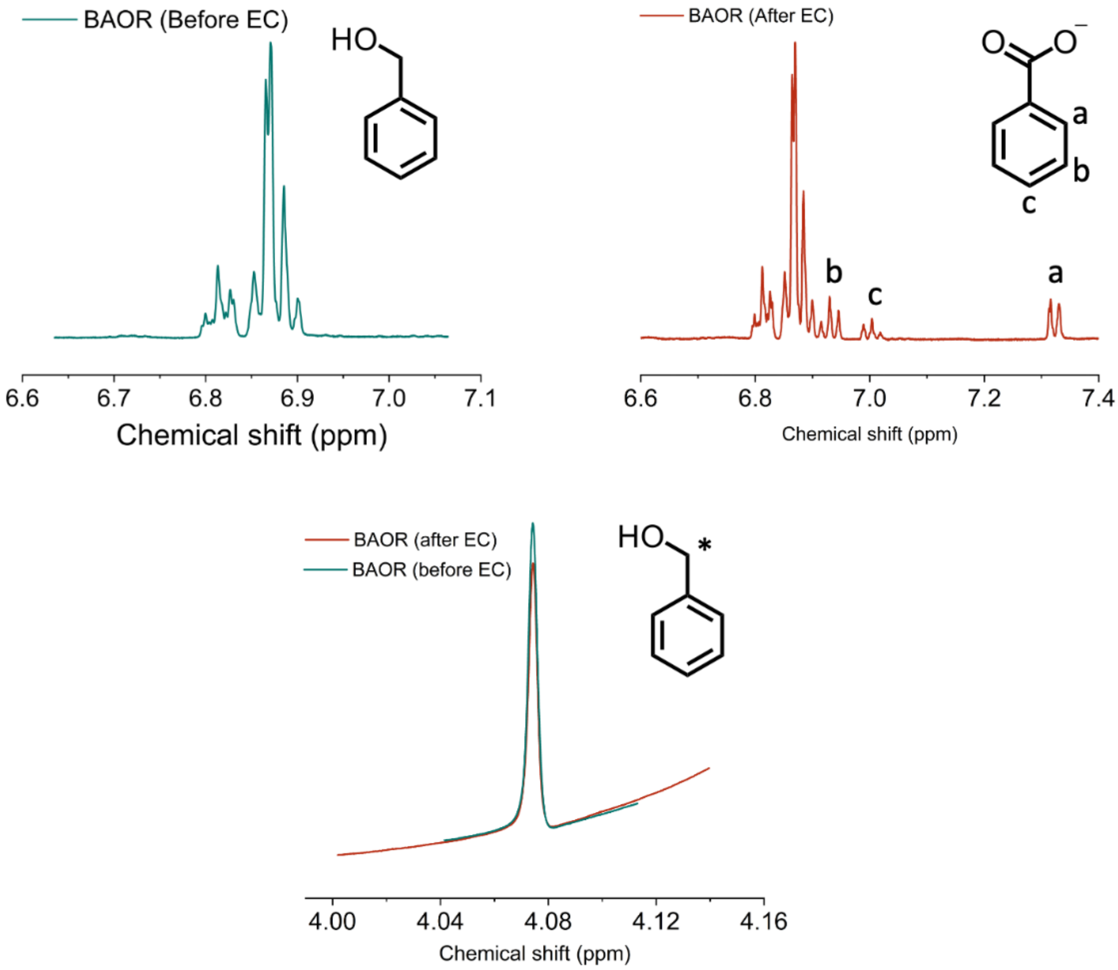

Figure S13. ^{1}H-NMR spectra of benzyl alcohol oxidation reaction (BAOR) before and after electrochemical (EC) treatment. The top-left spectrum shows benzyl alcohol prior to EC, with characteristic peaks from aromatic protons and the benzylic methylene group. After EC (top-right), the appearance of new aromatic resonances indicates the formation of benzoate. The bottom spectrum overlays the benzylic proton region before and after EC, confirming the loss of the alcohol signal upon oxidation.

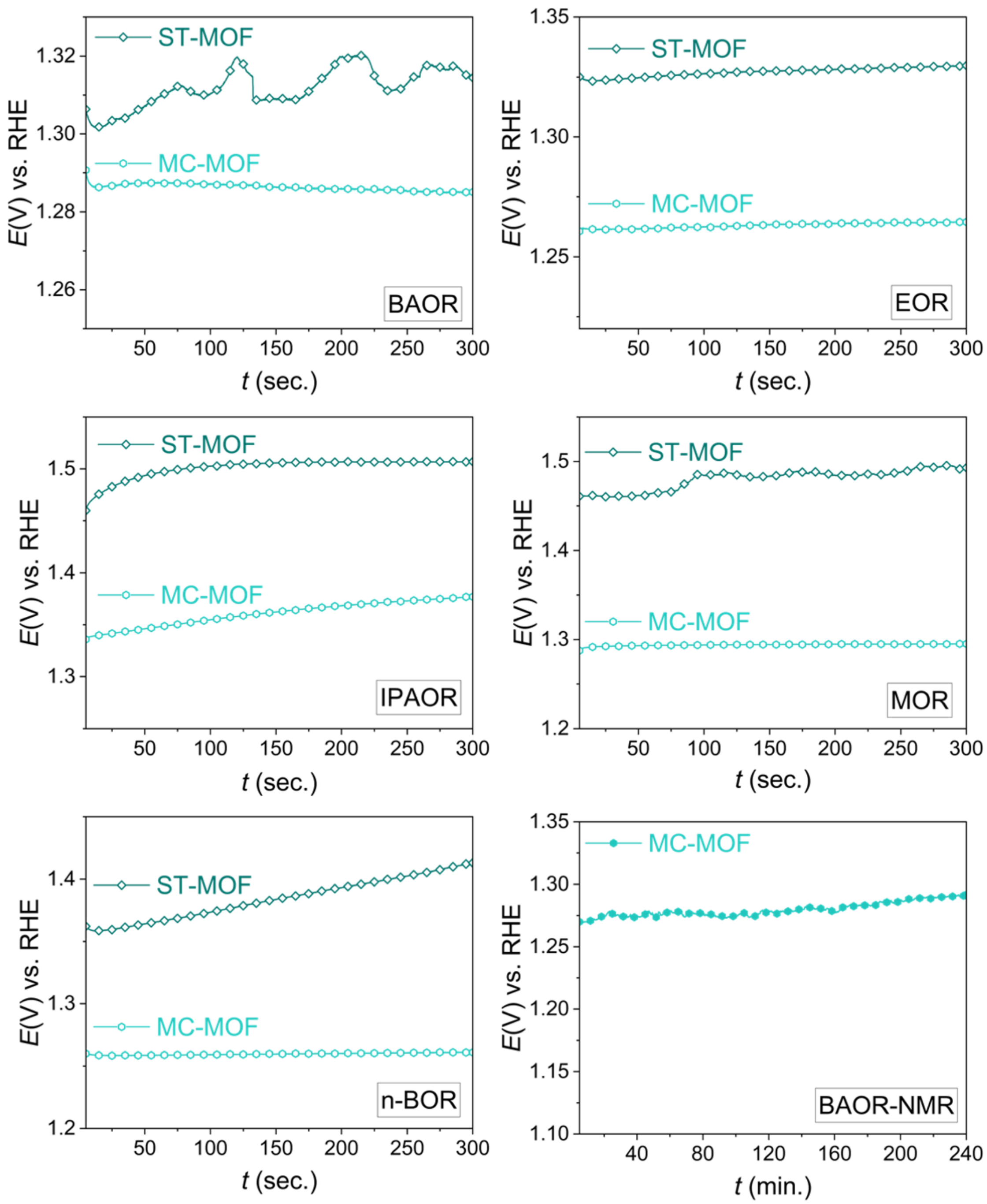

Figure S14. Chronopotentiometry profiles of different alcohol oxidation reactions recorded at a constant current density of 10 mA cm^{-2} on ST-MOF and MC-MOF electrocatalysts. The experiments include benzyl alcohol oxidation (BAOR), ethanol oxidation (EOR), isopropanol oxidation (IPAOR), methanol oxidation (MOR), and n-butanol oxidation (n-BOR).

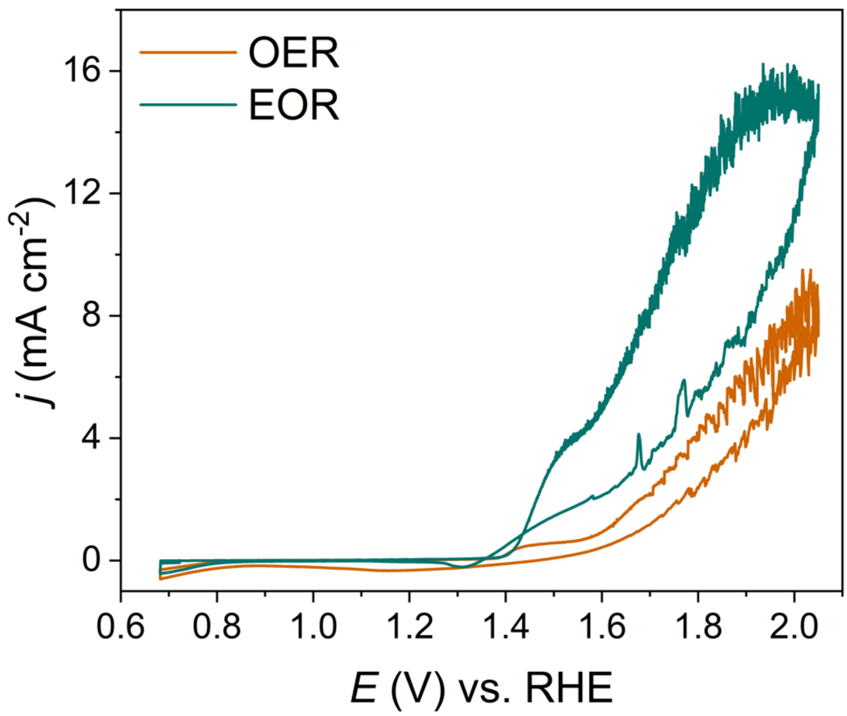

Figure S15. Cyclic voltammograms (CVs) of ethanol oxidation reaction (EOR) and oxygen evolution reaction (OER) recorded under operando conditions. The comparison highlights the lower onset potential and higher current density for EOR relative to OER, demonstrating the preferential oxidation pathway during electrochemical measurements.

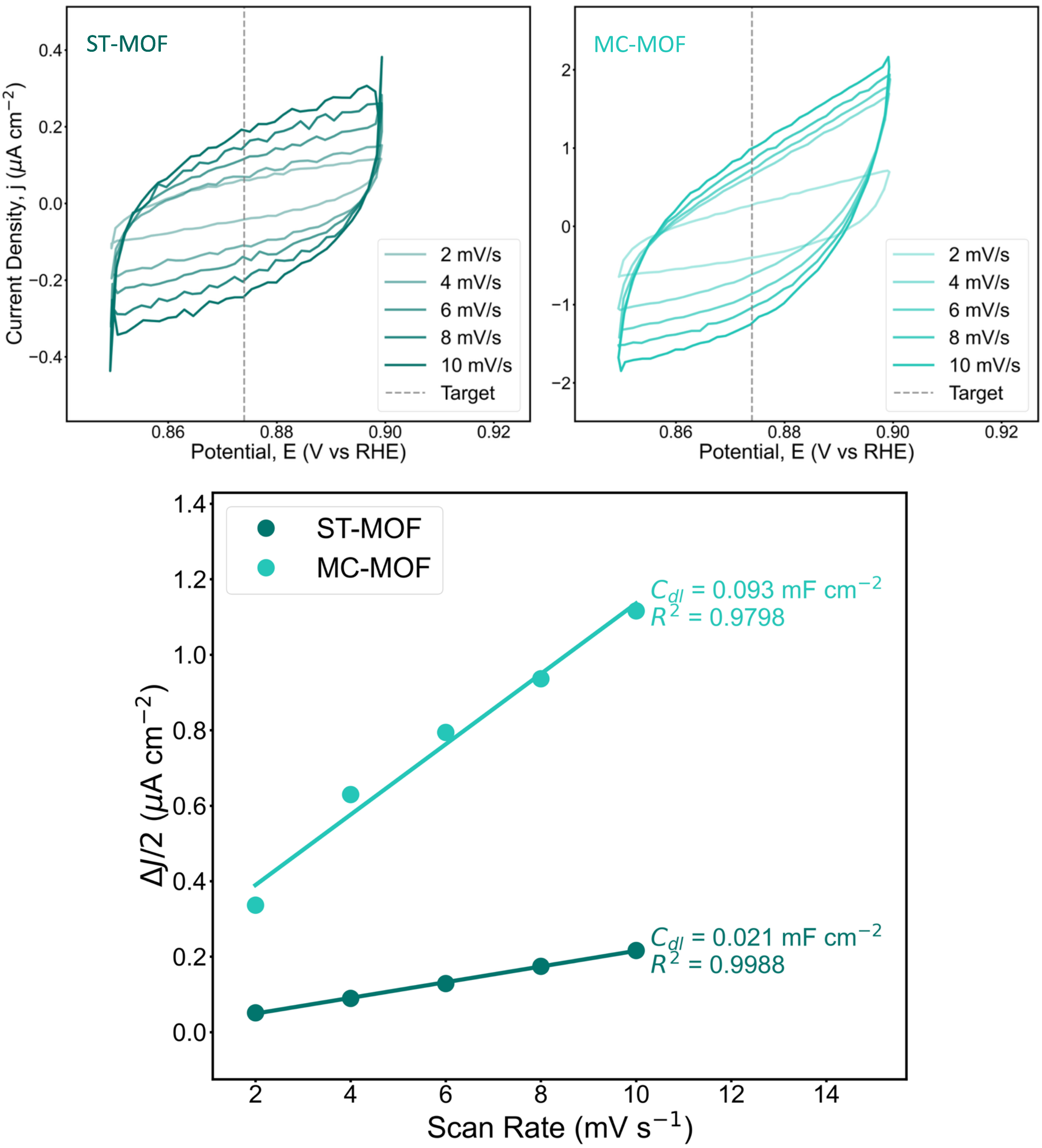

Figure S16. Evaluation of Electrochemically Active Surface Area (ECSA). (Top) Cyclic voltammograms (CVs) measured in the non-Faradaic potential region (0.85 to 0.9V vs. RHE) at various scan rates ranging from 2 to 10 mV $s^{-1}$for ST-MOF and MC-MOF. The vertical dashed line indicates the target potential (0.875V vs. RHE) where the capacitive current was extracted. (Bottom) Corresponding linear regression plots of the capacitive current density ($\Delta j/2$) as a function of the scan rate for both catalysts. The slope of the linear fit directly corresponds to the double-layer capacitance (C_{dl}). *Data presentation:* To confirm the reproducibility of the capacitive response, the cyclic voltammetry scans were repeated for (n = 5) consecutive cycles on the same electrode. The high goodness of fit is indicated by the R^2 values ($R^2 > 0.97$), and precluding the need for error bars or probability value testing.

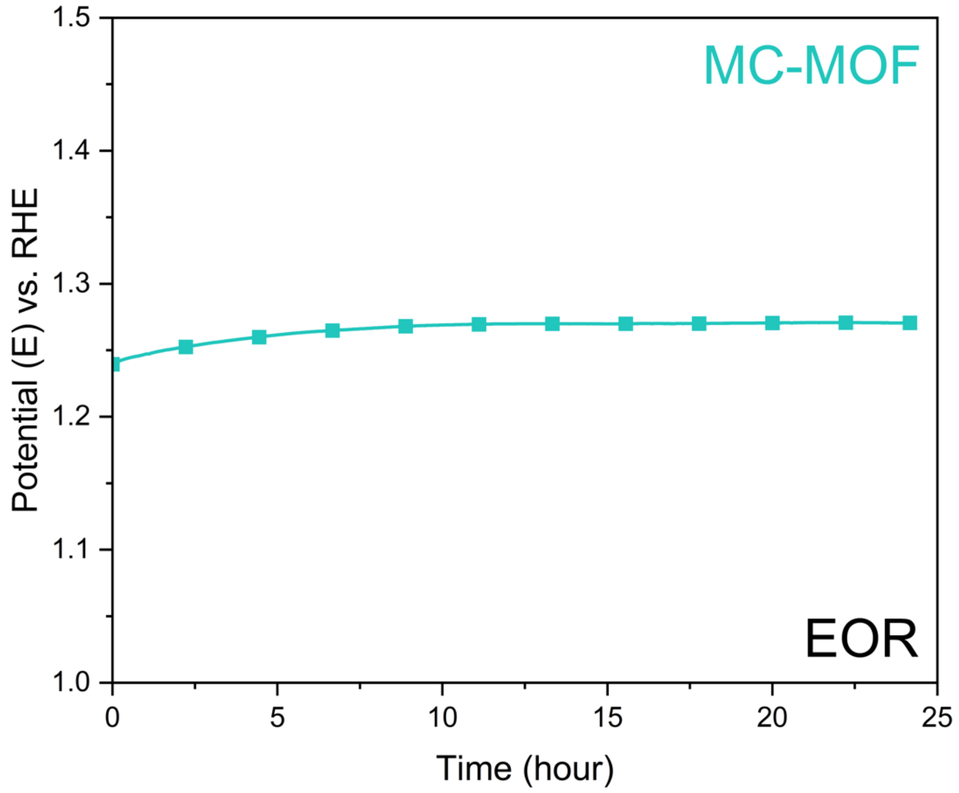

Figure S17. Long-term electrocatalytic stability of MC-MOF for the Ethanol Oxidation Reaction (EOR). Chronopotentiometry profile of the MC-MOF catalyst recorded over a 24-hour period at a constant applied geometric current density of 10 mA cm^{-2} The measurement was conducted in an electrolyte composition of 1M NaOH containing 0.1M ethanol. The steady potential response indicates the robust durability of the active sites under continuous anodic operation.

Experimental Techniques:

Single-Crystal X-ray Diffraction

Single-crystal X-ray diffraction (SCXRD) data for Ni-MOF were collected on a Bruker D8 Venture diffractometer equipped with graphite-monochromated Mo-Kα radiation (λ = 0.71073 Å). Data reduction and absorption corrections were performed using the SAINT and SADABS[1] software packages. Structures were solved by direct methods with SHELXT 2018 and refined by full-matrix least-squares on F^2 using SHELXL 2018, with anisotropic displacement parameters applied to all non-hydrogen atoms. Fourier and difference Fourier syntheses were employed in subsequent refinement cycles. Additional calculations were carried out using SHELXS (2018). The highly disordered guest molecules in the Ni-MOF structure, which contributed to elevated R and wR values, were treated using the SQUEEZE routine implemented in Olex2. A solvent-accessible void of approximately 150 $Å^3$ containing 24 electrons was identified and removed from the refinement. This residual electron density likely corresponds to approximately 2.4 water molecules, 1.3 methanol molecules, or a mixture of both. Due to severe disorder, these solvent molecules could not be modeled explicitly and were therefore accounted for using SQUEEZE. The final structure factors have been modified accordingly. Structural visualizations were generated using Mercury v4.0 and Diamond v4.6.5. Crystallographic data, refinement details, and key structural parameters for Ni-based MOF (Ni-MOF) are summarized in Table S1, S2.

Powder X-ray Diffraction

X-ray diffraction patterns were collected using Cu Kα radiation on a Bragg Brentano D8 Advanced diffractometer (Bruker AXS, Germany) equipped with a LYNXEYE XE-T detector. Samples were measured in reflection geometry in a 2θ range from 5° to 90° with a step size of 0.02° using a spinning setup.

Infrared Spectroscopy

The IR-spectra were recorded on a Bruker Vertex 70 spectrometer equipped with a Golden Gate diamond ATR (attenuated total reflectance) module.

Nuclear Magnetic Resonance

NMR experiments were performed on a 500 MHz NMR spectrometer system (VNMRS500, Varian Associates, Palo Alto, USA) operating at proton frequency of 499.9 MHz, which was equipped with a 5 mm OneNMR probe. Samples were studied without further modification or addition of deuterated solvent directly after filling into 5 mm NMR tubes. On each sample a proton spectrum with 16 scans was acquired at a pulse angle of 45° and a relaxation delay of 15 s.

Scanning Electron Microscopy

The Scanning Electron Microscopy (SEM) characterization was conducted on an XL 30 ESEM equipped with a tungsten cathode (FEI, Eindhoven, in 2020 electronic upgrade by point electronic GmbH). ESEM investigations were performed in "high-vacuum mode" of the microscope with an excitation voltage of 25kV. The signal was processed with a Secondary Electron detector (SE). Prior to the analysis all samples were coated with gold.

Electrochemical Measurements

Electrodes for linear sweep voltammetry were prepared by drop-casting an ink containing MC-MOF and ST-MOF powders onto a carbon-paper electrode. The catalyst ink was prepared by dispersing 5 mg of catalyst and 1 mg of Vulcan carbon black in 980 µL of a water/ethanol (1:1) mixture, followed by the addition of 20 µL of a 5 wt% Nafion solution. The mixture was then sonicated in an ultrasonic bath for 30 minutes. A carbon-paper electrode with a geometric area of 0.5*0.5 cm^2 was coated by drop-casting the ink to achieve a net catalyst loading of 50 µg cm^{-2}.

Electrochemical measurements were carried out in a three-electrode glass cell equipped with a Hg/HgO reference electrode and a platinum counter electrode, using a rotating disk electrode setup. The electrolyte was N_2-saturated 1.0 M NaOH (99.99% purity, Sigma-Aldrich). The potential was controlled with a Biologic VSP-3e potentiostat. Linear sweep voltammetry (LSV) for catalytic performance comparison and cyclic voltammetry (CV) for operando XAS experiment were conducted at scan rates of 5 mV s^{-1} and 1 mV s^{-1}, respectively. Ohmic losses were corrected by subtracting the uncompensated resistance (measured via high-frequency AC impedance), with 85% iR compensation applied. All measured potentials were converted to the reversible hydrogen electrode (RHE) scale using the relation: $E(V) = E_{ref} + E_{ref\ versus\ RHE}$, with $E_{ref\ versus\ RHE}$ the potential difference between the reference electrode and RHE.

X-ray Absorption Spectroscopy

Operando time-resolved X-ray absorption fine-structure (XAFS) spectroscopy at the nickel K-edge (8,333 eV) was carried out at the P64 beamline of the PETRA III synchrotron (Hamburg, Germany) in quick-XAFS mode using a Si(111) monochromator. The incident beam intensity was monitored with a nitrogen-filled ionization chamber, while additional chambers recorded transmission spectra of a nickel foil for energy calibration at the start of each scan. The X-ray beam size was kept below 2 mm × 2 mm.[3,4]

A custom-built single-compartment electrochemical cell was employed for operando measurements. Electrolyte was pumped through the cell at a flow rate of 0.4 ml min^{-1}. The Ni based MC-MOF were deposited on a Kapton foil with a layer of conductive carbon (DuPont Kapton 200RS100) and additionally sputter-coated in-house with a ~100nm layer of gold surrounding the area on which the catalyst was coated. For the XAS measurements in transmission mode, the catalyst concentration was was set to 8 mg/mL_{ink}. The catalyst was further sprayed on the gold coated Kapton substrate with a high loading of ~4-5 mg/cm^2. An Ag/AgCl electrode was used as the reference electrode, and platinum wire was used as counter electrode. Further, the flow of the electrolyte was controlled by a peristaltic pump operating at 0.4 mL/min, which also help to reduce the accumulation of the O_2 bubbles along the beam path. Finally, XAS measurements were acquired which performing a chronoamperometric technique at different applied potential. The applied potential was controlled using a Biologic

potentiostat. XAS data were extracted and calibrated with the JAQ software available at the P64 beamline. Subsequent processing and analysis of the Operando XAS spectra undertaken using a custom batch fitting python script utilizing the X-Ray Larch package.[5] Ex-situ data processing, analysis and fitting was performed according to the procedure described in ref. [6]

Operando Electrochemical Cell Design

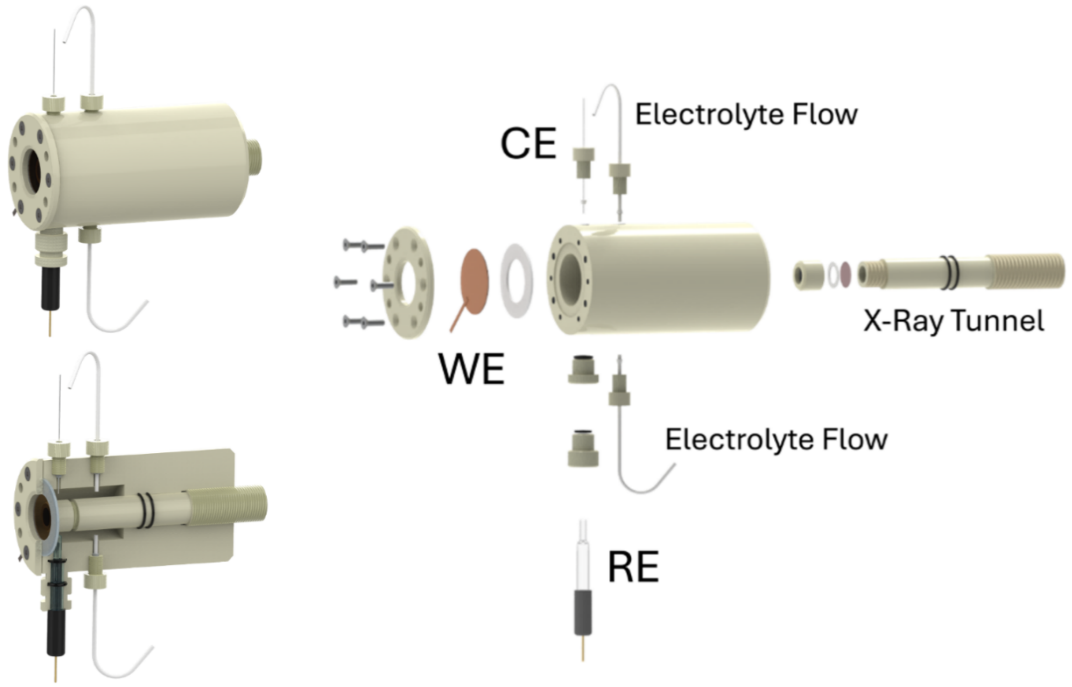

Figure S18. Dismantled and assembled view of an operando electrochemical cell designed for X-ray Absorption Spectroscopy (XAS) characterization.

This operando electrochemical cell is fabricated from PTFE (Polytetrafluoroethylene) to ensure chemical resistance and X-ray transparency. The cell configuration includes a working electrode (WE), counter electrode (CE), and reference electrode (RE), enabling standard three-electrode electrochemical measurements. The design allows electrolyte flow through dedicated inlets and outlets, maintaining controlled reaction conditions during operando experiments. Additionally, the cell features an adjustable distance between the X-ray window and the working electrode, providing flexibility to optimize the measurement and signal quality according to experimental requirements during XAS experiment.

Table S1. Crystallographic parameters of Ni-MOF

Compound Name	**Ni-MOF**
Temperature/K	150.00
Formula	$C_{17}H_{19}N_2NiO_7$
Formula Weight	422.05
Crystal System	Monoclinic
Space group	*C*2/c
a/Å	19.5287(5)
b/Å	9.6905(2)
c/Å	24.6993(6)
α/ ◦	90
β/ ◦	106.6660(10)
γ/ ◦	90
V/$Å^3$	4477.82(18)
Z	8
Dc/g cm^{-3}	1.252
μ/mm^{-1}	0.901
F(000)	1752.0
2θ range/ ◦	4.82 to 53.528
Reflections collected	41475
Independent reflections	4492
Data/restraints/parameters	4492/0/247
Goodness of fit (F^2)	1.068
R_1 (I ≥ 2σ(I))	0.0357
wR_2 (I ≥ 2σ(I))	0.0849
CCDC No.	2485713

Table S2. Selected bond lengths and bond angles in Ni-MOF

Ni-O4	2.0393(12)	Ni-O2	2.0960(13)
Ni -O1	2.0954(13)	Ni- O3	2.0799(14)
Ni-N2^a	2.0749(15)	Ni-N1	2.0683(16)
O4-Ni-O2	86.44(5)	O4-Ni-O1	81.80(5)
O4-Ni-O3	91.15(5)	O4-Ni-N2^a	172.93(6)
O4-Ni-N1	91.20(6)	O1-Ni-O2	88.73(5)
O3-Ni-O2	89.87(6)	O3-Ni-O1	172.88(5)
N2^a-Ni-O2	88.49(6)	N2^a-Ni-O1	93.16(6)
N2^a-Ni-O3	93.78(6)	N1-Ni-O2	177.28(6)
N1-Ni-O1	89.58(6)	N1-Ni-O3	91.55(7)
N1-Ni-N2^a	93.74(6)		

Symmetry: $a = -X, +Y, \frac{1}{2} - Z;$

Table S3. Measured MC-MOF and ST-MOF samples were fitted with structure obtained from the SCXRD measurement. Results from Ni K-edge EXAFS fitted parameters, including scattering path, coordination number (N), interatomic distance (R), inter atomic distance from the model (R_{eff}), Debey Waller factor (σ^2), amplitude reduction factor (S_O^2), energy shift parameter (ΔE), and R-factor, are given below. Finally, the total Ni-K edge EXAFS fit of MC-MOF and ST-MOF was also performed. Parentheses indicate a standard deviation of the last digit of fitted parameters. The Hanning window type, with dk = 1 is chosen to perform the Fourier transformation over the selected k-space range between 2 to 14 $Å^{-1}$. The fit to the model structure was performed between 1 and 3.3 Å.

<table>
<tr><th>Sample</th><th>Path</th><th>N</th><th>R(Å)</th><th>R_{eff}(Å)</th><th>σ² (Å²)</th><th>S_o²</th><th>ΔE (eV)</th><th>R-factor</th></tr>
<tr><td rowspan="8">ST-MOF</td><td>Ni-O</td><td>1</td><td>2.044(5)</td><td>2.03</td><td>0.007</td><td rowspan="8">1.1(1)</td><td rowspan="8">0.9(1)</td><td rowspan="8">0.019</td></tr>
<tr><td>Ni-N</td><td>2</td><td>2.077(5)</td><td>2.07</td><td>0.007</td></tr>
<tr><td>Ni-O</td><td>3</td><td>2.096(5)</td><td>2.09</td><td>0.007</td></tr>
<tr><td>Ni-H</td><td>6</td><td>2.7(1)</td><td>2.52</td><td>0.006</td></tr>
<tr><td>Ni-C</td><td>2</td><td>2.988(7)</td><td>2.98</td><td>0.006</td></tr>
<tr><td>Ni-C</td><td>3</td><td>3.017(7)</td><td>3.00</td><td>0.006</td></tr>
<tr><td>Ni-N,C</td><td>4</td><td>3.210(7)</td><td>3.20</td><td>0.006</td></tr>
<tr><td>Ni-N,C</td><td>4</td><td>3.217(7)</td><td>3.21</td><td>0.006</td></tr>
<tr><td rowspan="8">MC-MOF</td><td>Ni-O</td><td>1</td><td>2.04(1)</td><td>2.03</td><td>0.006</td><td rowspan="8">1.0(1)</td><td rowspan="8">1.2(1)</td><td rowspan="8">0.020</td></tr>
<tr><td>Ni-N</td><td>2</td><td>2.08(1)</td><td>2.07</td><td>0.006</td></tr>
<tr><td>Ni-O</td><td>3</td><td>2.10(1)</td><td>2.09</td><td>0.006</td></tr>
<tr><td>Ni-H</td><td>6</td><td>2.6(1)</td><td>2.52</td><td>0.007</td></tr>
<tr><td>Ni-C</td><td>2</td><td>2.987(6)</td><td>2.98</td><td>0.006</td></tr>
<tr><td>Ni-C</td><td>3</td><td>3.015(6)</td><td>3.00</td><td>0.006</td></tr>
<tr><td>Ni-N,C</td><td>4</td><td>3.208(6)</td><td>3.20</td><td>0.006</td></tr>
<tr><td>Ni-N,C</td><td>4</td><td>3.216(6)</td><td>3.21</td><td>0.006</td></tr>
</table>

Table S4. Comparison of the electrocatalytic performance of various MOF-based catalysts for the methanol oxidation reaction (MOR). While this table provides a general comparison of state-of-the-art materials, it is important to note that direct quantitative comparisons of current densities and overpotentials across different literature reports can be challenging. The observed electrocatalytic performance is highly dependent on several variable experimental parameters, particularly the concentration of methanol in the alkaline electrolyte and the specific type of working electrode substrate employed (e.g., glassy carbon, nickel foam, carbon cloth), stirring rate or flow rate, etc. Additionally, the catalyst mass loadings can inherently influence the reported geometric current densities. Therefore, this comparison is intended to contextualize the competitive performance of the MC-MOF within the broader landscape of recently reported systems.

Catalyst	Potential (V)	Current / Current Density (mA cm^{-2})	Electrolyte	Ref.
MoS_2@CoNi-ZIF	1.24 (vs. RHE)	430.08 mA g^{-1}	1.0 M KOH	[7]
NiIr-MOF/NF	1.41 (vs. RHE)	100 mA cm^{-2}	1.0 M KOH	[8]
PdNPs@ZIF-67-Corannulene/GCE	0.4 (vs. Ag/AgCl)	90.20 mA cm^{-2}	1.0 M KOH	[9]
$CoxNi_{3-x}(HAB)_2$ MOF-2	1.6 (vs. RHE)	92.80 mA cm^{-2}	1.0 M KOH	[10]
Ni-BTC/4 wt % rGO	1.66 (vs. RHE)	200.02 mA cm^{-2}	1.0 M NaOH	[11]
NiCo-MOF	1.6 (vs. RHE)	225 mA cm^{-2}	1.0 M KOH	[12]
rGO/NiO-MOF	0.8 (vs. Ag/AgCl)	275.85 mA cm^{-2}	1.0 M NaOH	[13]
Zr-MOF@PANI/Ni-NPs	0.75 (vs. Ag/AgCl)	291.8 mA cm^{-2}	1.0 M NaOH	[14]
4 wt% rGO-FeO/NiO MOF	0.85 (vs. Ag/AgCl)	486.14 mA cm^{-2}	1.0 M NaOH	[15]
MC-MOF	1.36 (vs. RHE)	50 mA cm^{-2}	1.0 M NaOH	This work

Note S1. Qualitative Product Analysis

Following the 4-hour chronopotentiometry experiments, the resulting alkaline electrolytes were analyzed using ^{1}H NMR spectroscopy to identify the primary liquid-phase oxidation products. Through this method, we successfully identified acetate, acetone, butyrate, and benzoate as the dominant oxidation products for ethanol, isopropanol, n-butanol, and benzyl alcohol, respectively. It should be noted that the current electrochemical cell setup and available analytical facilities do not permit the reliable capture, detection, or quantification of volatile or gaseous byproducts, such as CO and CO_2. Consequently, establishing a rigorous quantitative carbon mass balance and calculating exact Faradaic efficiencies fall outside the scope of the present study. The ^{1}H NMR data presented herein (Figures S10–S13) serve strictly as qualitative confirmation of the substrate dependent reaction pathways facilitated by the Ni-MOF catalysts.

Note S2. Relationship Between Synthesis Methodology, Morphology, and Catalytic Performance

As demonstrated by our structural and electronic characterizations (XRD, XPS, and XANES), the local coordination environment and oxidation state (+2) of the Ni centers remain identical regardless of whether the MOF is synthesized via mechanochemical (MC) or solvothermal (ST) routes. Because the intrinsic nature of the active sites is unchanged, the enhanced geometric electrocatalytic activity of the MC-MOF is directly linked to its distinct morphology-specifically, the formation of smaller, highly agglomerated particles that expose a greater density of accessible catalytic sites to the electrolyte.
If an alternative synthesis protocol-such as a heavily modulated solvothermal approach using specific solvent molecule or capping agents-were engineered to produce the similar nano-agglomerated morphology, it is highly probable that the resulting electrocatalytic performance would be comparable. However, a distinct advantage of the mechanochemical approach is that it inherently and rapidly yields this high-surface-area morphology without requiring complex solvent engineering or additional directing agents.
Furthermore, the mechanochemically induced morphology is highly tunable. By systematically varying controllable milling parameters-such as the milling frequency (Hz), total milling time, and the volume of the liquid-assisted grinding (LAG) solvent -the particle size, defect density, and agglomeration state can be further modified. For instance, increasing the milling frequency imparts greater kinetic energy, which can lead to smaller crystallite sizes and a higher density of defect sites, directly influencing catalytic turnover. While a mapping of these mechanochemical parameters falls outside the scope of this investigation, it represents a critical avenue for future catalyst optimization.

Note S3. Comparative XANES Analysis of MC-MOF under Initial Conditions

To further elucidate the catalyst's electronic behavior in the reaction environment prior to active electrocatalysis, XANES spectra were compared for three specific states: the pristine as-synthesized MC-MOF, the catalyst immersed in 1.0 M NaOH, and the catalyst immersed in 1.0 M NaOH containing 0.1 M ethanol. As shown in Figure S19, the white line intensities and absorption edge positions for all three conditions remain virtually identical. This spectral alignment confirms that the initial electronic environment and the +2-oxidation state of the Ni centers are highly stable upon contact with both the highly alkaline electrolyte and the alcohol substrate. The introduction of the catalyst to the reaction medium does not induce spontaneous oxidation to higher valence states (such as Ni^{3+}or Ni^{4+}); these

higher oxidation states are only accessed dynamically upon the application of an anodic potential during the operando electrochemical measurements.

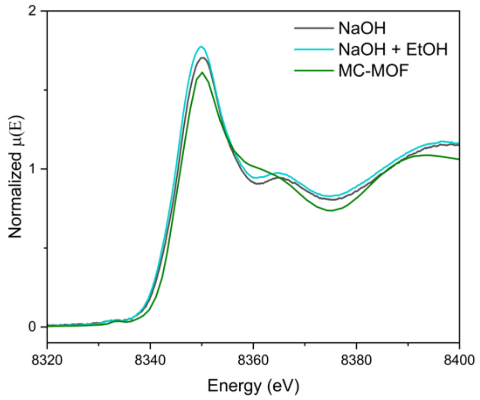

Figure S19. Normalized Ni K-edge XANES spectra comparing the pristine MC-MOF catalyst, the catalyst in 1 M NaOH, and the catalyst in 1 M NaOH with ethanol.

Note S4. Proposed Reaction Mechanism for Alcohol Oxidation

Based on our electrochemical data, product identification (^{1}H NMR), and operando XAS observations, we propose a generalized reaction mechanism for alcohol oxidation on the Ni-centers of the MC-MOF catalyst. Figure S20 illustrates this pathway using the ethanol oxidation reaction (EOR) as a representative model.

The catalytic cycle initiates with the adsorption of the alcohol onto the active site, displacing a coordinated water molecule, followed by deprotonation in the alkaline medium to form a Ni^{2+}-alkoxy intermediate. Subsequent proton-coupled electron transfer (PCET) steps generate transient high-valent Ni^{3+} species, which rapidly facilitate the dehydrogenation of the substrate to yield a carbonyl intermediate (an aldehyde for primary alcohols; a ketone for secondary alcohols) via a 2-electron oxidation process. At this stage, the metal center returns to its stable Ni^{2+} resting state. For secondary alcohols (e.g., Isopropanol), the reaction naturally terminates at this 2-electron oxidation stage, yielding the corresponding ketone (acetone), as the lack of an α-proton prevents further oxidation under these conditions. For primary alcohols (e.g., Ethanol, n-Butanol, Benzyl alcohol), the transient aldehyde intermediate undergoes further nucleophilic attack by hydroxide ions from the electrolyte. Subsequent PCET steps drive a second 2-electron oxidation, ultimately yielding the fully oxidized carboxylate product (acetate, butyrate, and benzoate, respectively) via a complete 4-electron pathway. Importantly, the dominance of stable Ni^{2+} intermediates depicted throughout this catalytic cycle perfectly aligns with our operando Quick-XAS data. Because the high-valent Ni^{3+} states are highly transient and rapidly reduced by the alcohol substrate, the Ni^{2+} states accumulate as the resting phase and thus dominate the millisecond-timescale X-ray absorption spectra.

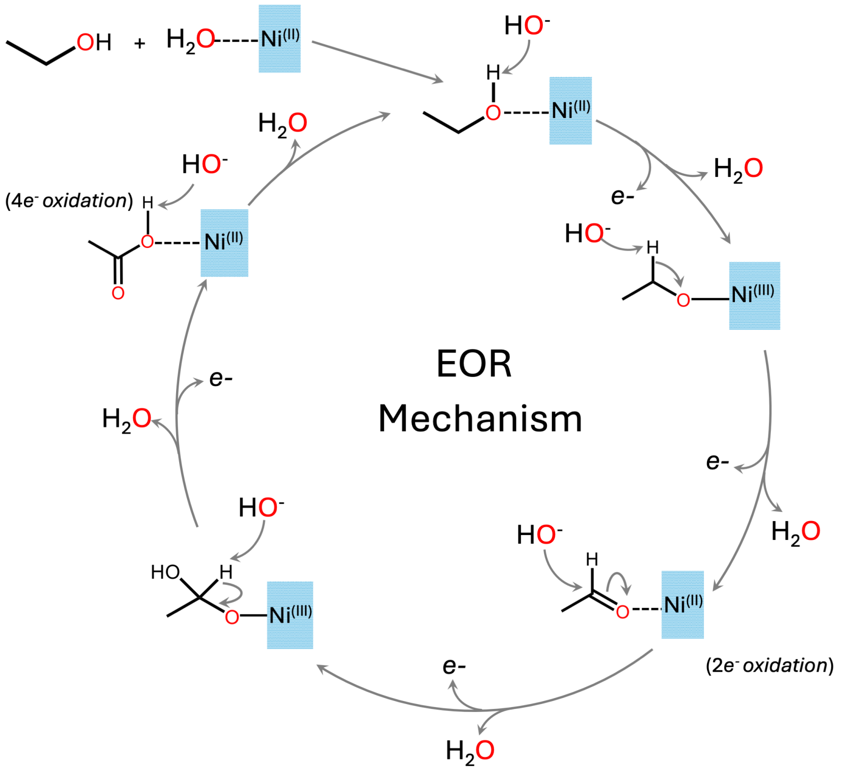

Figure S20. Proposed reaction mechanism for the electrocatalytic oxidation of alcohols on the MC-MOF catalyst, using the Ethanol Oxidation Reaction (EOR) as a representative model. The schematic highlights the transient high-valent Ni species and the stable Ni^{2+} intermediates throughout the 2-electron and 4-electron oxidation pathways.

References:

[1] G. M. Sheldrick, *Acta Crystallogr A Found Crystallogr* **2008**, *64*, 112.

[2] G. M. Sheldrick, *Acta Crystallogr C Struct Chem* **2015**, *71*, 3.

[3] W. A. Caliebe, V. Murzin, A. Kalinko, M. Görlitz, Taipei, Taiwan, **2019**, p. 060031.

[4] B. Bornmann, J. Kläs, O. Müller, D. Lützenkirchen-Hecht, R. Frahm, Taipei, Taiwan, **2019**, p. 040008.

[5] M. Newville, *J. Phys.: Conf. Ser.* **2013**, *430*, 012007.

[6] A. Roy, S. Kumar, A. Guilherme Buzanich, C. Prinz, E. Götz, A. Retzmann, T. Hickel, B. Bhattacharya, F. Emmerling, *Advanced Materials* **2024**, *36*, 2408114.

[7] Y. Liu, B. Hu, S. Wu, M. Wang, Z. Zhang, B. Cui, L. He, M. Du, *Applied Catalysis B: Environmental* **2019**, *258*, 117970.

[8] Y. Xu, M. Liu, M. Wang, T. Ren, K. Ren, Z. Wang, X. Li, L. Wang, H. Wang, *Applied Catalysis B: Environmental* **2022**, *300*, 120753.

[9] H. Khuntia, K. S. Bhavani, T. Anusha, T. Trinadh, M. C. Stuparu, P. K. Brahman, *Colloids and Surfaces A: Physicochemical and Engineering Aspects* **2021**, *615*, 126237.

[10] B. Cui, C. Wang, S. Huang, L. He, S. Zhang, Z. Zhang, M. Du, *Journal of Colloid and Interface Science* **2020**, *578*, 10.

[11] L. Yaqoob, T. Noor, N. Iqbal, H. Nasir, N. Zaman, *Catalysts* **2019**, *9*, 856.

[12] M. Wang, C. Wang, L. Zhu, F. Rong, L. He, Y. Lou, Z. Zhang, *Applied Catalysis A: General* **2021**, *619*, 118159.

[13] T. Noor, N. Zaman, H. Nasir, N. Iqbal, Z. Hussain, *Electrochimica Acta* **2019**, *307*, 1.

[14] S. Sheikhi, F. Jalali, *Fuel* **2021**, *296*, 120677.

[15] T. Noor, M. Mohtashim, N. Iqbal, S. R. Naqvi, N. Zaman, L. Rasheed, M. Yousuf, *Journal of Electroanalytical Chemistry* **2021**, *890*, 115249.
